# Study of Factors Affecting UV-Induced Photo-Degradation in Different Types of Polyethylene Sheets

**DOI:** 10.3390/polym16192709

**Published:** 2024-09-25

**Authors:** Bochu Du, Chenghao Lee, Ying Ji

**Affiliations:** Research Centre for Resources Engineering towards Carbon Neutrality, Research Institute for Intelligent Wearable Systems, Hong Kong Polytechnic University, Kowloon, Hong Kong SAR, China; bochudu@polyu.edu.hk (B.D.); chenghao.lee@polyu.edu.hk (C.L.)

**Keywords:** oxo-degradation, photo-degradation, polyethylene, pro-oxidants, plastic sheets

## Abstract

Enhancing the degradability of polyethylene plastics could provide a potential solution to the overwhelming crisis of plastic waste. Conventional studies have focused on the degradation of polyethylene thin films. This study investigated UV-induced photo-degradation according to ASTM D5208-14 in polyethylene sheets with thicknesses ranging from 0.4 to 1.2 mm. The impacts of sample thickness, metal pro-oxidants, polyethylene resin types and foaming were explored through the characterization of the carbonyl index, molecular weight, tensile properties and crystallinity. As pro-oxidants, single iron or manganese stearate demonstrated a concentration-dependent trend in accelerating the photo-degradation of polyethylene sheets. The thickness, foaming and resin type—such as low-density polyethylene (LDPE) and high-density polyethylene (HDPE)—significantly impacted the rate of photo-oxidation. Thick polyethylene sheets (1.2 mm) exhibited a heterogenous and depth-dependent degradation profile. As the photo-degradation progressed, the enhanced crystallinity, reduced UV transmittance and formation of crosslinks were able to prevent further oxidative cleavage of the polyethylene chain. This study investigated the time course and factors affecting the photo-degradation of polyethylene sheets, which could provide insights into the formulation design of photo-degradable polyethylene plastics.

## 1. Introduction

The polyolefins consumed in our daily life primarily consist of commodity plastics, which refers to plastic products that are produced in high volume and rapidly turned over. The most commonly applied polyethylene phenotypes include low-density polyethylene (LDPE) and high-density polyethylene (HDPE), labeled as symbol 4 and symbol 2 in the International Resin Identification Code (RIC), respectively. Polyethylene has been manufactured into a broad range of commodity products, including plastic bags, packaging films or foams, bottles and containers for foods or household items, etc. LDPE and HDPE accounted for 30 to 40% of municipal plastic waste according to statistics obtained before the COVID-19 pandemic [1,2]. Since the outbreak of COVID-19, more consumers have switched to single-use products due to the primary concern of sanitation. In addition, social distancing and related restrictions increase customer dependency on online shopping, which boosts the consumption of plastic packaging materials [3]. As COVID-19 could become a long-term threat to be managed, this sharp rise in the production, consumption and disposal of polyethylene commodity products is expected to persist in the long run. It is reasonable to predict that proper handling of plastic waste could become a critical challenge in the upcoming years.

Although gravity separation strategies—such as the vibrating, screening and sink float methods—could be utilized for plastic waste recycling [4,5], polyethylene has a density range (0.88 to 0.96 g/cm^3^) overlapping with those of other plastic wastes such as polypropylene and expanded polystyrene, which challenges efficient sorting. The additives and fillers in plastic also introduce complexity in density-based waste sorting. Furthermore, polyethylene consists of multiple phenotypes, including LDPE, HDPE, linear low-density polyethylene (LLDPE), co-polymers with polypropylene, etc. Each phenotype resin varies in its molecular weight, melt flow index and crystallinity. The identification and isolation of polyethylene phenotypes suitable for the manufacturing of different recycled products further adds to the difficulties. Lastly, sanitation concerns that the recycling of commodity plastics may lead to health hazards (as potential carriers of pathogens) collectively challenge the recycling of polyethylene wastes [6]. Moreover, the incineration of plastic waste can release persistent organic pollutants and toxins that are still managed as municipal solid waste and can overload the landfill capacity [7]. At the current stage, technological barriers still exist that prevent the efficient recycling or energy conversion of polyethylene waste. The persistence of synthetic plastic in the natural environment and the exhausting of landfill space emphasize the importance of sustainable strategies for plastic waste management.

As an alternative approach, enhancing the biodegradability of polyethylene could provide a potential solution to the overwhelming waste issue [8]. The biodegradation of polyolefin involves an enzymatic fragmentation stage and a bio-assimilation stage. Enzymatic fragmentation is initiated by the breakdown of polyolefin chains into smaller segments. Subsequently, microorganisms engulf the low-molecular-weight fragments to achieve the complete conversion of polyolefins into gases, water, minerals and biomass [9]. Koutny et al. [10] reported that an average molecular weight of under 5000 Da was required to achieve effective biodegradation. A molecular weight of under 5000 Da was also considered as a prerequisite for biodegradation in tiered, criteria-based standards, such as ASTM D6954-18 [11], for evaluating the biodegradability of plastics. However, the huge molecular weight (tens to hundreds of kDa) of post-consumer polyethylene waste could be particularly challenging for polyolefin-degrading microorganisms to achieve fragmentation. The hydrophobic nature of polyethylene further reduces the attachment and anchorage of microorganisms, hindering extracellular enzymatic breakdown and subsequent intracellular bio-assimilation [12]. To address this challenge, oxo-degradation could provide an effective approach to obtain fragmented polyethylene with a reduced molecular weight, as the oxidized functional groups enhance the interfacial hydrophobic/hydrophilic balance and facilitate the attachment and biodegradative action of microorganisms.

The oxo-degradation of polyethylene occurs in disposal environments, induced by exposure to thermal or light irradiation. Oxo-degradation can lead to effective fragmentation and a molecular weight decrease for polyethylene waste. The photolytic generation of oxygen-containing free radicals (including macro-alkoxy radicals, hydroxyl radicals, etc.) propagates the oxidative cleavage of the polyethylene chain. Oxo-degradation introduces functional groups—including aldehydes, ketones, carboxylic acids, esters and vinylene—via β scission, the abstraction of hydrogen, cage reactions and Norrish reactions [13]. Pro-oxidants such as metal stearates accelerate the initiation of photo-oxidation by the rapid generation of alkyl macroradicals [14]. Meanwhile, the decomposition of alkyl hydroperoxides has been catalyzed by metal stearates to accelerate the cleavage of polyethylene chains [15]. Metal stearates have demonstrated efficient pro-oxidant effects at minor concentrations [15] and can be explored as plastic additives without impacting the manufacturing process or the performance of polyethylene products. Although oxo-degradation contributes to the rapid disintegration of polyethylene, the potential issue of microplastics cannot be ignored. If fragmentation only occurs at the macroscopic level, without an effective molecular weight decrease, polyethylene can persist in disposal environments, followed by its infiltration into the microstructure of ecosystems [16]. Therefore, this study explores the process of polyethylene photo-oxidation into low-molecular-weight fragments, which is important in ASTM D6954-18 [11] to evaluate biodegradability.

Research has been performed to investigate the performance of transition metal carboxylates, photocatalytic nanoparticles and commercial pro-oxidants in accelerating the oxo-degradation of polyethylene plastics [17,18,19,20,21,22]. However, conventional studies on the oxo-degradation profile of polyethylene have primarily focused on thin films, with an average thickness of less than 0.25 mm. In addition to film products, polyethylene has been widely applied in plastic containers, lids, packaging foams, tubing, fitting, etc. In contrast to those through thin film, the limited penetration of light irradiation and the potentially restricted oxygen transport through thick specimens can lead to different degradation profiles. In this study, the photo-degradation of polyethylene sheets with average thicknesses varying from 0.4 to 1.2 mm is investigated under ASTM D5208-14 [23] UV weathering conditions. Tier 1 evaluations of ASTM D6954-18 are performed by exploring the tensile properties, molecular weight, carbonyl index and degree of crystallinity. The impacts of the sample thickness, type and concentration of metal stearate, resin type, foaming structure, etc., are investigated to assess the photo-degradability of polyethylene sheets.

## 2. Materials and Methods

### 2.1. Materials

Low-density polyethylene (LDPE, with a density of 0.92 g/cm^3^ and a melt flow index (MFI) of 2 g/10 min, tested at 190 °C and 2.16 kg) and high-density polyethylene (HDPE, with a density of 0.95 g/cm^3^ and a MFI of 2 g/10 min) resins were provided by Sinopec Group (Maoming, China). The LDPE resin contained 0.10 wt% Irganox 1010 and 0.33 wt% Irgafos 168. The HDPE resin contained 0.08 wt% Irganox 1010 and 0.18 wt% Irgafos 168. Both resins were free of UV stabilizers. Iron (III) stearate (FeSt) was purchased from Aladdin Chemical Reagent Co., Ltd (Shanghai, China). Manganese (II) stearate (MnSt) was purchased from Santa Cruz Biotechnology (Santa Cruz, CA, USA). Expancel^®^ 093DU120 expandable microspheres were supplied from Akzo Nobel (Sundsvall, Sweden).

### 2.2. Preparation of Polyethylene Sheets

The metal stearates (5 wt%) were first mixed with LDPE or HDPE resin and compounded into masterbatches on a Haake MiniCTW twin screw compounder (Thermo Fisher Scientific, Karlsruhe, Germany). The metal stearate masterbatch was uniformly mixed with polyethylene resin in a high-speed mixer at 60 rpm. The manufacturing of polyethylene sheets was performed on a bench-top single-screw extruder (Hapro Electronic Groups, Harbin, China); the diameter of the screw was 20 mm and the length-to-diameter ratio (L/D) was 25:1. The single-screw extruder was equipped with a flat die head (60 mm in width and 1.5 mm in thickness). The temperatures for the extrusion were 110 °C, 130 °C, 150 °C and 160 °C from feed throat to die. The extruded polyethylene melt was pressed by a stainless-steel roller and collected on a conveyor belt for natural cooling to room temperature. The thickness of the polyethylene sheets was controlled by adjusting the screw speed (50 to 150 rpm) and the collection speed (0.5 to 3 m/min). The smooth surface of the as-manufactured polyethylene sheet in direct contact with the roller was labeled the “face” side, and the opposite matte surface was labeled the “back” side. The formulations of the polyethylene sheets are summarized in Table 1. To confirm the metal concentrations, the as-prepared polyethylene sheets were microwave digested with nitric acid; the concentrations of Fe and Mn were then detected by inductively coupled plasma mass spectrometry on a Neptune Plus ICP-MS (Thermo Fisher Scientific, Karlsruhe, Germany).

### 2.3. Accelerated UV Weathering

The UV weathering of polyethylene sheets was performed in an accelerated weathering chamber equipped with UVA-340 lamps (QUV, Q-Lab, Westlake, OH, USA), following ASTM D5208-14 [23]. Continuous UV irradiation at 340 nm was delivered with a constant irradiance of 0.89 W/m^2^. The uninsulated black panel temperature was controlled at 50 °C. The face sides of the polyethylene sheets (150 mm in length and 25 mm in width) were positioned towards the UVA-340 lamps. As listed in Table 1, the thickness of each polyethylene sheet was uniform and within ± 10% of the nominal thickness (0.4 mm, 0.8 mm or 1.2 mm). The UV irradiance and temperature at the sample site were continuously monitored. At the end of the pre-defined weathering durations, the photo-degraded samples were collected for the following studies.

### 2.4. Morphological Characterization

To investigate the time-dependent morphological changes that occurred in the surface and cross-section of polyethylene sheets during photo-degradation, samples were collected before weathering and 2 to 8 weeks post accelerated weathering. The collected samples were gold-sputtered and observed on a scanning electron microscope (JSM-6490, Jeol, Tokyo, Japan) at 20 kV.

### 2.5. Tensile Test

Polyethylene sheets were cut into standard dog bone specimens (Type V), and tensile testing was performed following ASTM D638-14 [24] and ASTM D3826-18 [25]. For each polyethylene formulation, 5 specimens were tested as replicates. The specimens were exposed to the same UV weathering conditions indicated in Section 2.3. Tensile testing was performed on an Instron 5566 universal testing system (Instron, Norwood, MA, USA) equipped with a video extensometer and a 500 N load cell. The strain rate was set constant at 10 mm/min. The elongation of the specimen was monitored by the video axial strain. According to ASTM D3826-18 [25], the polyethylene sheets were considered to be degraded to the brittle point when 75% or more of the 5 tested specimens exhibited an elongation at break of ≤5%. The specimens that already fractured during exposure were considered to have reached the brittle point and were not subjected to the tensile test.

### 2.6. ATR-FTIR and UV Spectroscopy

Attenuated total reflection Fourier-transform infrared spectroscopy (ATR-FTIR) was performed on a Spectrum100 spectrometer (PerkinElmer, Waltham, MA, USA). The FTIR absorption spectra before and after UV weathering were recorded for both the face and back sides of the samples. The carbonyl index (CI) was calculated from the ratio between the integrated peak area of the carbonyl absorption peak (C=O) from 1850 to 1670 cm^−1^ and that of the methylene (-CH_2_-) scissoring peak from 1500 to 1420 cm^−1^, following a previously published study with slight modification [26]. Triplicates were tested for each group, and the CI is expressed as mean values with standard deviations. The UV transmittance (T%) was tested on a spectrometer supplemented with a 150 mm double-beam-mode integrating sphere (PerkinElmer, Waltham, MA, USA).

$$CI=\frac{Peak\;area(1850-1670\;{cm}^{-1})\;}{Peak\;area(1500-1420\;{cm}^{-1})}$$

### 2.7. WAXD and DSC Characterization

The changes in the crystallinity of polyethylene sheets after photo-degradation were characterized by wide angle X-ray diffraction (WAXD) on an Aeris XRD device (Malvern PANalytical, Worcestershire, UK), operated at 40 kV and 15 mA with Cu Kα radiation. The scanning rate was 5°/min and the range of 2θ was from 5° to 50°. The peaks corresponding to the amorphous region and orthorhombic unit cell structure of planes (110) and (200) in polyethylene crystallites were fitted, and the degree of crystallinity (*Xc*) was calculated from the peak intensities (*I*_110_, *I*_200_ and *I_a_*) via the following equation [27].

$$Xc=\frac{I_{110}+1.42I_{200}}{I_{110}+1.42I_{200}+0.68I_{a}}$$

The thermal properties were characterized via differential scanning calorimetry (DSC) on a Mettler TGA/DSC3+ thermal analyzer (Mettler Toledo, Zurich, Switzerland). The samples were analyzed over the temperature range from ambient to 250 °C in a nitrogen atmosphere, with a programmed heating rate of 10 °C /min. The area of the endothermic melting peak was compared before and after UV weathering.

### 2.8. Detection of Molecular Weight and Distribution

Polyethylene samples were dissolved in 1,2,4-trichlorobenzene stabilized with 125 mg/L of butylated hydroxytoluene (BHT) at 150 °C. The solution was filtered and tested via high-temperature gel permeation chromatography (HT-GPC) on an Infinity II HT-GPC system (Agilent, Palo Alto, CA, USA) with three PLgel MIXED-B LS (300 × 7.5 mm) columns. A refractive index detector was utilized, and the retention times of polyethylene samples were calibrated against polystyrene standards. The intrinsic viscosity [η] was calculated from the molecular weight (MW) by the Mark–Houwink equation ([η] = KMW^α^). A universal calibration curve was established between Log (MW[η]) and the retention time. As per the manufacturer’s instruction, the parameters K and α for polymers in trichlorobenzene were as follows: for polystyrene, K = 12.1 × 10^−5^ dL/g and α = 0.707; for polyethylene, K = 40.6 × 10^−5^ dL/g and α = 0.725. The molecular weight range of polyethylene that followed universal calibration was from 3.0 × 10^5^ Da to 1.3 × 10^3^ Da. The weight-average molecular weight (${\bar{M}}_{w}$), number-average molecular weight (${\bar{M}}_{n}$) and dispersity of the polyethylene were tested and compared with the initial molecular weight before weathering (${\bar{M}}_{w0},\;{\bar{M}}_{n0}$). The concentrations of crosslinks and chain scission after UV weathering were calculated based on the following equations [28].

$$\mathrm{Chain}\;\mathrm{scission}=\frac{4}{3}(\frac{1}{{\bar{M}}_{n}\;}-\frac{1}{{\bar{M}}_{n0}})-\frac{2}{3}(\frac{1}{{\bar{M}}_{w}}-\frac{1}{\;{\bar{M}}_{w0}})$$

$$\mathrm{Crosslink}=\frac{1}{3}(\frac{1}{{\bar{M}}_{n}\;}-\frac{1}{{\bar{M}}_{n0}})-\frac{2}{3}(\frac{1}{{\bar{M}}_{w}}-\frac{1}{\;{\bar{M}}_{w0}})$$

### 2.9. Fungal Incubation

The fungal incubation was performed according to ISO 846:2019 [29]. Spores of *P. chrysosporium* (ATCC 24725) after 7 days of culture on potato dextrose agar were collected and adjusted to a concentration of 1 × 10^6^/mL. The spore suspension was inoculated into potato dextrose broth (Sigma-Aldrich, St. Louis, MO, USA) at a dilution ratio of 1/100. Pre-weighed, disinfected polyethylene sheets after 4 or 8 weeks of UV weathering were transferred into the culture medium. The inoculated culture was incubated for 4 weeks at 30 °C. After incubation, the polyethylene sheets were washed with 0.1 M phosphate buffer to remove loosely attached fungi and were subsequently fixed with 2.5% glutaraldehyde in 0.1 M phosphate buffer for 24 h at 4 °C, followed by gradient dehydration in a series of ethanol aqueous solutions (50%, 70%, 80%, 90% and 100%). The fixed polyethylene samples were dried overnight, gold-sputtered and observed on a scanning electron microscope. For the weight loss study, polyethylene sheets were repetitively washed with 0.1 M phosphate buffer and 70% ethanol to remove as much fungal mass as possible. The sheets were then dried at 40 °C until no further weight changes occurred, and the sample weight was measured. Triplicates of polyethylene sheets were tested in each group.

### 2.10. Statistical Analysis

The data are presented as mean values with standard deviations. All data were analyzed using one-way ANOVA, followed by Tukey’s multiple comparison tests, and *p* < 0.05 indicated statistical significance.

## 3. Results and Discussion

### 3.1. Time Course of UV-Induced Photo-Degradation of Polyethylene Sheets

A series of pristine and pro-oxidant-containing polyethylene sheets were incubated under UV weathering conditions (continuous UV irradiation at 0.89 W/m^2^ @ 340 nm, 50 °C) as defined by ASTM D5208-14 [23]. The polyethylene resins, types and concentrations of metal stearate pro-oxidants, thicknesses and sample abbreviations are listed in Table 1. The pro-oxidants investigated in this study included iron stearate (FeSt) and manganese stearate (MnSt). The concentrations of metals in the polyethylene sheets were confirmed using ICP-MS before UV weathering. To investigate the time course of UV-induced photo-degradation, representative formulations were tested, including pristine polyethylene (LDPE-0.8) with an average thickness of 0.8 mm and polyethylene containing 30 ppm of Fe (LDPE-0.8-Fe30) with equivalent thickness. Characterizations of the morphology, carbonyl index, molecular weight and tensile properties were performed on both the unweathered samples and those subjected to 2, 4, 6 or 8 weeks of weathering.

**Figure 1:**
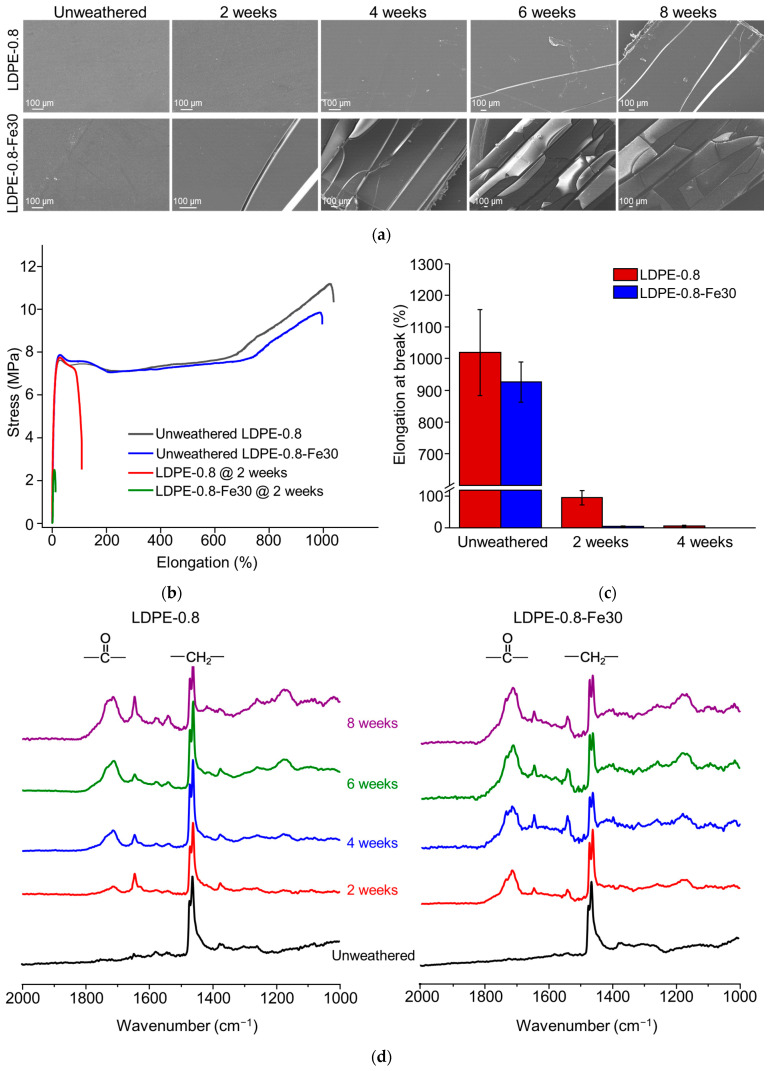

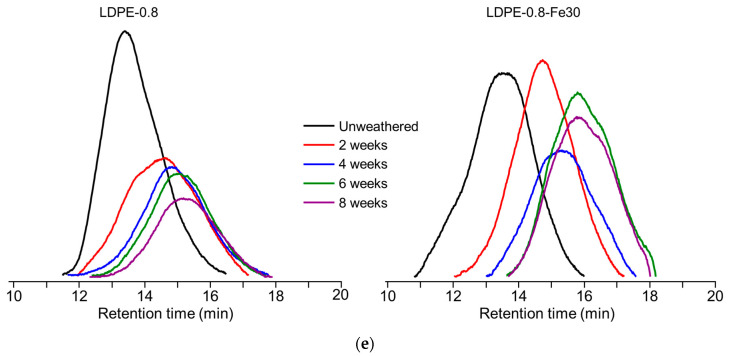
Time course of UV-induced photo-degradation in polyethylene sheets. (**a**) SEM morphological characterization of LDPE-0.8 and LDPE-0.8-Fe30 after 2, 4, 6 and 8 weeks of UV weathering. (**b**) Tensile curves of LDPE-0.8 and LDPE-0.8-Fe30 before and after 2 weeks of UV weathering. (**c**) Elongation at break of photo-oxidized polyethylene sheets detected in the tensile test. (**d**) ATR-FTIR spectra of LDPE-0.8 and LDPE-0.8-Fe30 to characterize the development of carbonyl peaks. (**e**) HT-GPC chromatographs of photo-oxidized LDPE-0.8 and LDPE-0.8-Fe30.

The SEM morphological characterization results are shown in Figure 1a. For pristine LDPE-0.8, noticeable cracks developed after 4 to 6 weeks of weathering. In the pro-oxidant-containing LDPE-0.8-Fe30, accelerated crack formation was observed after just 2 weeks of weathering. Furthermore, LDPE-0.8-Fe30 exhibited significant embrittlement after 6 or 8 weeks of weathering. These results are consistent with a previous report [30] that described observed substantial crack development in recycled LDPE after 1000 h of UV-induced photo-oxidation. The formation of cracks during polyethylene degradation could be a collective result of chain cleavage with a loss of strength and ductility, as well as increased consumption of the tie molecules between crystalline regions during the photo-oxidation process [31].

The tensile properties were studied before and after photo-degradation. As shown in Figure 1b,c, unweathered LDPE-0.8 exhibited an average elongation at break of 1020.2% ± 135.5%. After 2 and 4 weeks of weathering, the elongation at break for LDPE-0.8 decreased significantly to 95.6% ± 22.5% and 5.8% ± 2.9%, respectively. In the case of LDPE-0.8-Fe30, the deterioration of tensile performance was further accelerated, with the elongation at break reduced from 925.8% ± 63.2% to 3.9% ± 1.5% after just 2 weeks of weathering. LDPE-0.8-Fe30 after 4 weeks of weathering fractured before the tensile test, indicating that it had already reached the brittle point (elongation at break ≤5%). In contrast, pristine polyethylene sheets required more than 4 weeks of weathering to reach the brittle point. For all the pro-oxidant-containing polyethylene sheets investigated in this study (data not specified), 2 weeks of weathering resulted in brittleness that met the degradation endpoint defined by ASTM D3826-18 [25]. For consistency with previous studies [32,33], the key criterion for embrittlement was the loss of 95% elongation at break. Scission of tie molecules occurred, preventing the transfer of tensile stress to the neighboring lamellar crystals, which led to the embrittlement of photo-oxidized LDPE. Compared to the molecular weight and CI, the tensile performance of the polyethylene sheets demonstrated greater sensitivity to UV weathering. Significant embrittlement in the polyethylene sheets was observed after 2 to 4 weeks of weathering, while a decrease in ${\bar{M}}_{w}$ to ≤5000 Da required a weathering duration exceeding 8 weeks.

The CIs for both the face side (facing toward UV lamps during weathering) and the back side (facing away from UV lamps during weathering) were characterized. Table 2 summarizes the CI_face_ and CI_back_ values of LDPE-0.8 and LDPE-0.8-Fe30 over the time course of UV-induced photo-degradation. Consistent with the development of carbonyl peaks in the FTIR spectra, both CI_face_ and CI_back_ demonstrated a growing trend from 2 weeks to 8 weeks of weathering (Figure S1, Supporting Information). At each weathering time point, LDPE-0.8-Fe30 exhibited CI values greater than those of LDPE-0.8, which correlated with the pro-oxidant effect of FeSt that accelerated the oxidative cleavage of the polyethylene chain.

ATR-FTIR characterization was performed to identify changes in surface functional groups during photo-degradation. The penetration depth for ATR-FTIR typically falls into the μm range; samples with a thickness exceeding this range require additional consideration of the different surfaces. As shown in Figure 1d, the FTIR spectra of polyethylene over the time course of photo-degradation (0 to 8 weeks) demonstrated a growing carbonyl peak from 1670 cm^−1^ to 1850 cm^−1^. This carbonyl peak could include oxidized functional groups such as carboxylic acids (1700 cm^−1^), carboxylate (1550 cm^−1^, not included in the CI calculation), ketones (1714 cm^−1^), lactones (1780 cm^−1^), esters and aldehydes (1733 cm^−1^) [34]. In comparisons of pro-oxidant-containing polyethylene sheets (LDPE-0.8-Fe30) with pristine polyethylene (LDPE-0.8), a distinct carbonyl peak was observed for LDPE-0.8-Fe30 from 2 weeks onward, and the carbonyl peak area for LDPE-0.8-Fe30 was consistently greater than that for LDPE-0.8 at each time point. In addition to the carbonyl peak, a vinyl peak was observed from 1650 to 1660 cm^−1^, which correlated with the C = C stretching vibration. As reported by Gardette et al. [13], the photo-degradation of polyethylene could be initiated by the generation of macro-alkoxy and hydroxyl radicals. The β-scission of the macro-alkoxy radical can cleave the polyethylene chain and form aldehydes. Furthermore, the cage reaction between macro-alkoxy and hydroxyl radicals could introduce ketones to the polyethylene chain, which could induce the Norrish photolytic reaction, resulting in vinyl unsaturation or chain-end ketones. Both aldehydes and chain-end ketones could react to form carboxylic acids, esters or lactones. Therefore, the development of carbonyl and vinyl groups (Figure 1d) indicated the scission of the polyethylene chain.

The molecular weight detection results are summarized in Table 2 and Figure 1e. For LDPE-0.8-Fe30, a significant decrease in ${\bar{M}}_{w}$ was detected, from 2.07 × 10^5^ Da (unweathered) to 5.63 × 10^3^ Da (6 weeks). However, the molecular weight decrease plateaued after 6 weeks of weathering, with an ${\bar{M}}_{w}$ value of 5.44 × 10^3^ Da (8 weeks). Pristine LDPE-0.8 exhibited a slower rate of molecular weight decrease, and the ${\bar{M}}_{w}$ value was reduced from 2.08 × 10^5^ Da (unweathered) to 1.45 × 10^4^ Da (8 weeks). Throughout the tested weathering duration, neither pristine LDPE-0.8 nor pro-oxidant-containing LDPE-0.8-Fe30 reached the degradation endpoint (${\bar{M}}_{w}$ ≤ 5000 Da) defined in ASTM D6954-18 (Tier 1) [11]. When the molecular decrease for the LDPE sheets (Table 2) was compared with that for polyethylene thin film [35] (<20 μm) under equivalent weathering conditions, the polyethylene film exhibited a more rapid decrease in molecular weight (>95% molecular weight decrease after 14 days of photo-oxidation). This difference in the rate of molecular weight decrease suggested that thickness significantly impacted the photo-degradation of polyethylene.

### 3.2. Effect of Thickness on the Photo-Degradation of Polyethylene Sheets

Polyethylene sheets with different thicknesses (0.4 mm, 0.8 mm and 1.2 mm) were exposed to UV weathering for 4 or 8 weeks. The molecular weights of the polyethylene sheets were detected and are summarized in Table 3 and Figure S2 (Supporting Information). LDPE-1.2 exhibited a significantly delayed decrease in ${\bar{M}}_{w}$ compared to LDPE-0.8 and LDPE-0.4. After 8 weeks of weathering, the ${\bar{M}}_{w}$ value of LDPE-1.2 was 3.75 × 10^4^ Da, and the ${\bar{M}}_{w}$ value of LDPE-0.4 was 7.55 × 10^3^ Da. The results indicated that the rate of molecular weight decrease was inversely correlated with the thickness of the polyethylene sheets.

**Figure 2:**
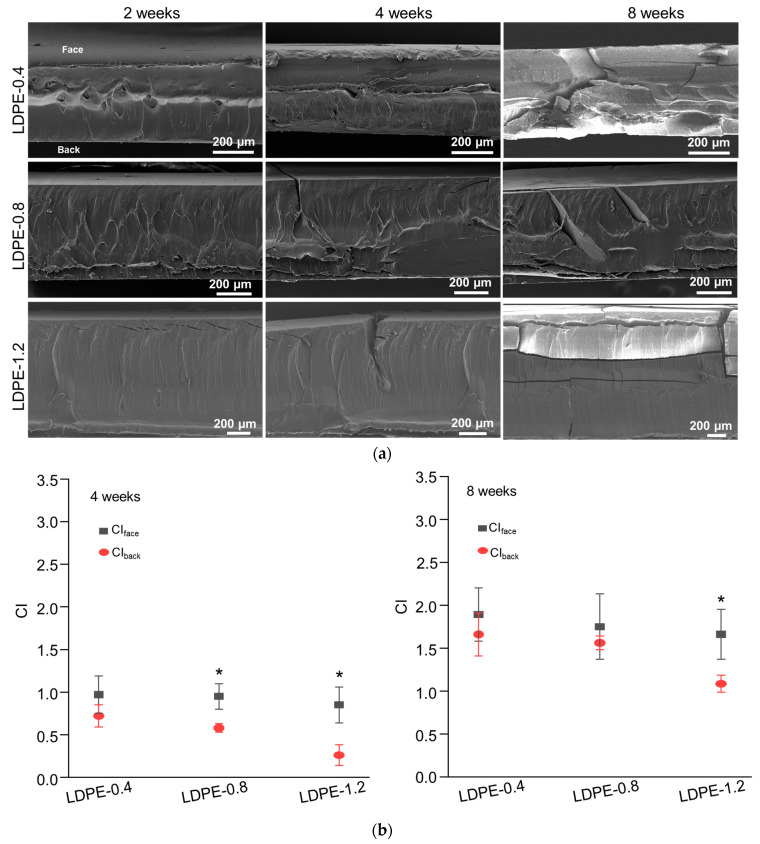

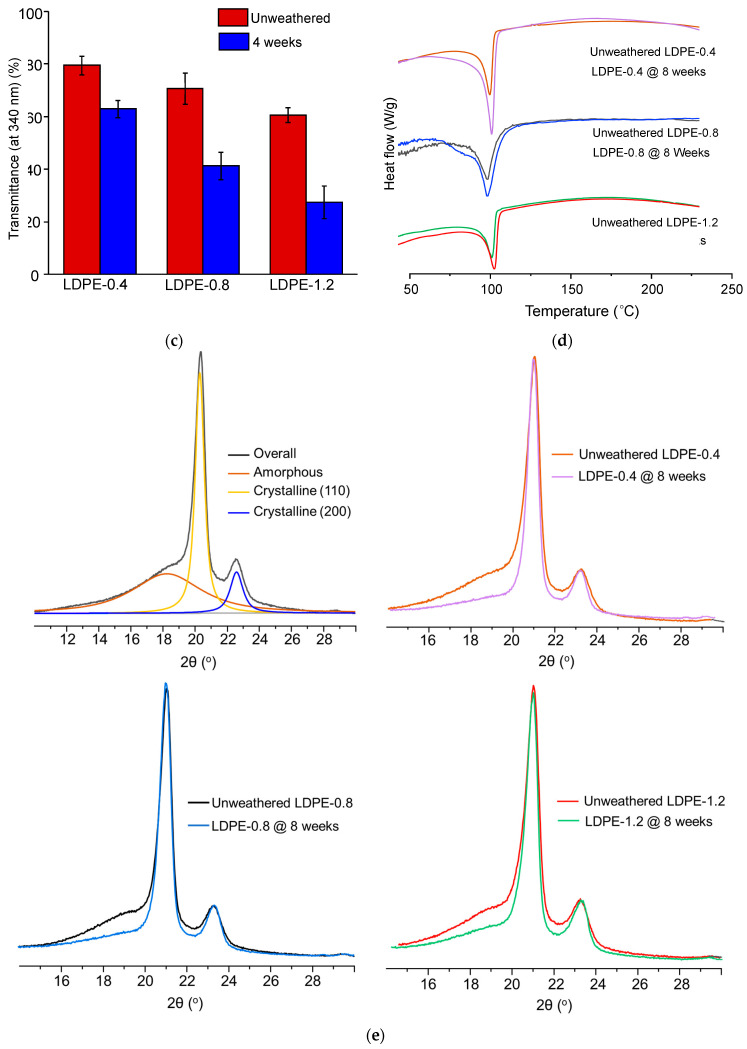

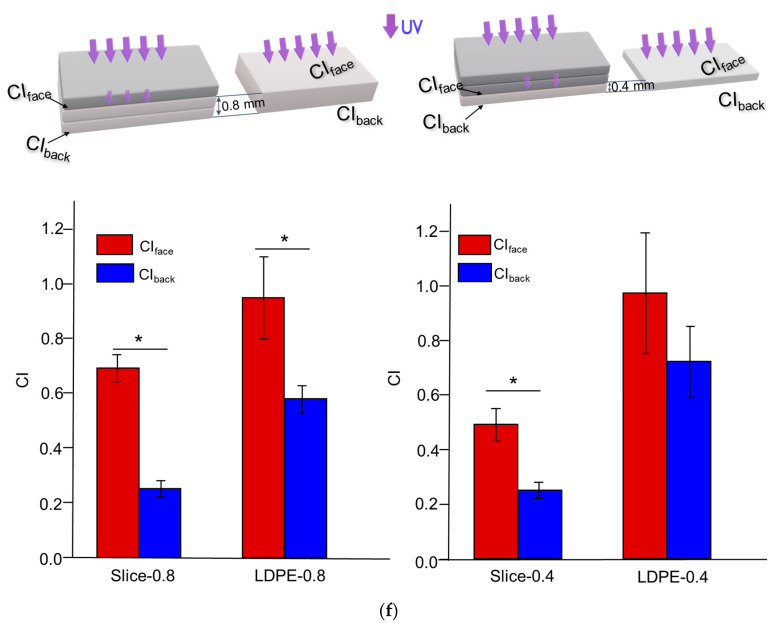
Impact of thickness (0.4 mm, 0.8 mm and 1.2 mm) on the photo-degradation of polyethylene sheets after 4 or 8 weeks of UV weathering. (**a**) SEM characterization of the cross-sections of photo-oxidized polyethylene sheets. (**b**) Detection of CI_face_ and CI_back_. (**c**) UV transmittance of polyethylene sheets before and after 4 weeks of weathering. (**d**) DSC curves of polyethylene sheets after 8 weeks of UV weathering. Endothermic melting peaks were compared. (**e**) WAXD spectra of polyethylene sheets before and after 8 weeks of UV weathering. Peak fitting demonstrated the presence of an amorphous peak and crystalline peaks corresponding to the (110) and (200) planes in polyethylene crystallites. (**f**) Depth-dependent CI profile in photo-oxidized LDPE sheets. Three slices in 400 μm increments were sectioned from oxidized LDPE-1.2 after 4 weeks of UV weathering. CI values from slices at different distances to the UV-irradiated upper face were detected. As indicated by arrows, CI_face_ and CI_back_ values detected in the second and third slices (abbreviated as Slice-0.8) were compared with those for unsliced LDPE-0.8. CI values from the third slice (abbreviated as Slice-0.4) were compared with those for unsliced LDPE-0.4. * indicates statistical significance (*p* < 0.05).

In the SEM characterization of the cross-sectional morphology (Figure 2a), uniform deterioration and crack development were observed in LDPE-0.4 as photo-oxidation progressed. In the case of LDPE-1.2, cracks originated from the face side and extended downwards, indicating a heterogenous photo-oxidation rate in the thick polyethylene sheet. By week 4, the cracks in LDPE-1.2 were not fully developed compared to those in thinner LDPE samples, which could be correlated with the critical ${\bar{M}}_{w}$ value for ductile-to-brittle transition. The critical ${\bar{M}}_{w}$ value for this transition is reported to be in the range of 4 to 10 × 10^4^ Da [36]. The ${\bar{M}}_{w}$ value of LDPE-1.2 at week 4 was 8.63 × 10^4^ Da, which did not exceed the ${\bar{M}}_{w}$ threshold for complete embrittlement. The delayed increase in *Xc* in LDPE-1.2 at week 4 (Table 3) suggested that the remaining tie chains in the amorphous region could impede crack formation.

The CI_face_ and CI_back_ values of polyethylene sheets after 4 and 8 weeks of UV weathering are displayed in Figure 2b. At each time point, the CI_face_ values of LDPE sheets with different thicknesses exhibited a similar trend of increase. To the contrary, a dependence on thickness was observed for CI_back_. For LDPE-0.4, no significant difference was observed between CI_face_ and CI_back_ after either 4 or 8 weeks of UV weathering. For LDPE-0.8, a significantly smaller CI_back_ value was detected after 4 weeks; however, this difference disappeared after 8 weeks of weathering. In contrast, CI_back_ was consistently smaller than CI_face_ for LDPE-1.2 after both 4 and 8 weeks of weathering, indicating a potentially delayed oxidative rate for the thicker LDPE sheet (1.2 mm) at the back side.

The tested LDPE sheets were partially transparent when unweathered and gradually became fully opaque during UV weathering. The UV transmittance before and after 4 weeks of weathering is characterized in Figure 2c. Unweathered LDPE sheets exhibited thickness-dependent transmittance at 340nm. LDPE-1.2 exhibited reduced transmittance at 340 nm (60.5% ± 2.9%) compared to LDPE-0.8 (70.6% ± 5.9%) and LDPE-0.4 (79.4% ± 3.5%). Furthermore, reduced UV transmittance was observed in all photo-oxidized LDPE sheets, indicating that photo-oxidation could introduce a barrier that prevents the penetration of UV irradiation.

The DSC characterization corroborated the increase in crystallinity after photo-oxidation, as evidenced by the rise in the melting peak area observed in all tested LDPE sheets after 8 weeks of UV weathering (Figure 2d). The melting peak of LDPE-0.8 was determined from 118.5 °C to 119.7 °C, with no significant change in the melting peak position after weathering. However, a distinct increase in the melting peak area was detected after weathering, demonstrated by a melting enthalpy of 163.2 J/g in unweathered LDPE-0.8 that increased to 259.8 J/g after 8 weeks of weathering. The crystallinity of photo-oxidized LDPE sheets was characterized by DSC and WAXD. In the WAXD spectra of unweathered LDPE-0.8 (Figure 2e), crystalline peaks corresponding to the orthorhombic unit cell structure of the (110) and (200) planes in polyethylene crystallites were identified within the 2θ ranges of 20.5° to 21° and 22.7° to 23.5°, respectively. An amorphous peak was identified within the 2θ range of 18.5° to 19.5°. After 8 weeks of weathering, a significant reduction in the intensity of the amorphous peak was noted in LDPE-0.8, suggesting that the photo-degradation of polyethylene could enhance crystallinity. A similar trend of reduction in the amorphous peak was observed in LDPE sheets with thicknesses of 0.4 mm and 1.2 mm (Figure 2e). After 8 weeks of weathering, the *Xc* value of LDPE-0.4 increased from 67.5% to 85.9%, and the *Xc* value of LDPE-1.2 increased from 54.6% to 80.4% (Table 3). In the LDPE sheets with different thicknesses, the increases in *Xc* after 8 weeks were all statistically significant when compared to unweathered LDPE samples.

Previous research demonstrated the importance of oxygen diffusion in contributing to the oxidation rates of thick polymer samples [37]. The consumption of oxygen during oxidative reactions can deplete the oxygen supply, resulting in a depth-dependent, heterogeneous degradation profile in thick samples. To analyze the potentially heterogeneous degradation profile of polyethylene sheets after UV weathering, LDPE-1.2 was selected due to the statistically significant difference between its CI_back_ and CI_face_ values (Figure 2b). LDPE-1.2 after 4 weeks of UV weathering was sectioned into three slices at 400 μm increments. The CI was characterized for each slice and compared with the CIs detected in unsliced samples. As shown in Figure 2f, the CIs in sliced samples exhibited a decreasing trend that was inversely related to the distance to the UV-irradiated upper face, as reflected by the CI_back_ and CI_face_ values detected in Slice-0.8 and Slice-0.4. In addition, compared to those for unsliced LDPE-0.8, significantly reduced CI_back_ and CI_face_ values were observed in Slice-0.8, and this trend was also noted in the comparison between Slice-0.4 and unsliced LDPE-0.4 (Figure 2f). This depth-dependency of the CI could be correlated with oxygen accessibility and UV transmittance in different slices.

The relative oxygen concentration of each slice in photo-oxidized LDPE-1.2 was calculated based on the model developed by Cunliffe et al. [38], as detailed in Tables S2 and S3 (Supporting Information). As shown in Figure S3 (Supporting Information), the distribution of the oxygen concentration exhibited an asymmetrical profile. The UV-irradiated upper face was expected to have maximum accessibility to both oxygen and UV irradiance, while at the unexposed back, maximum oxygen accessibility but minimal UV irradiance was expected. With the use of CI as the indicator of oxidation progress, it should be noted that the depth-dependent profile of CI diverged from the depth-dependent oxygen distribution (Figure S3, Supporting Information). However, the depth dependency of photo-oxidation progress in LDPE-1.2 showed a correlation with the change in UV transmittance (Figure 2c). This result indicated that the impact of UV penetration at different depths of the samples could potentially outweigh the influence of oxygen accessibility during the early to middle stages of UV weathering.

### 3.3. Effect of Pro-Oxidant Metals on the Photo-Degradation of Polyethylene Sheets

In this study, FeSt and MnSt were selected as the representative pro-oxidants. The Fe or Mn concentration in the tested polyethylene sheets was determined as 30 ppm or 60 ppm, and the thickness of the samples was maintained at 0.8 mm. When the Fe concentration exceeded 60 ppm, the polyethylene sheets exhibited a significant orange hue, which could adversely affect the appearance of plastic products.

The changes in ${\bar{M}}_{w}$ in FeSt- or MnSt-containing polyethylene sheets are summarized in Table 4. Both FeSt and MnSt exhibited a concentration-dependent effect in accelerating photo-degradation. Significantly reduced ${\bar{M}}_{w}$ values were detected for LDPE-0.8-Fe60 (${\bar{M}}_{w}$ = 8.92 × 10^3^ Da after 4 weeks and ${\bar{M}}_{w}$ = 5.05 × 10^3^ Da after 8 weeks) compared to LDPE-0.8-Fe30 (${\bar{M}}_{w}$ = 1.32 × 10^4^ Da after 4 weeks and ${\bar{M}}_{w}$ = 5.44 × 10^3^ Da after 8 weeks). A similar concentration-dependent trend was observed with MnSt. In comparisons of Fe and Mn at equivalent concentrations, MnSt resulted in a less efficient pro-oxidant effect, as demonstrated by the delayed decrease in ${\bar{M}}_{w}$ in LDPE-0.8-Mn60 (${\bar{M}}_{w}$ = 2.21 × 10^4^ Da after 4 weeks and ${\bar{M}}_{w}$ = 1.02 × 10^4^ Da after 8 weeks).

**Figure 3:**
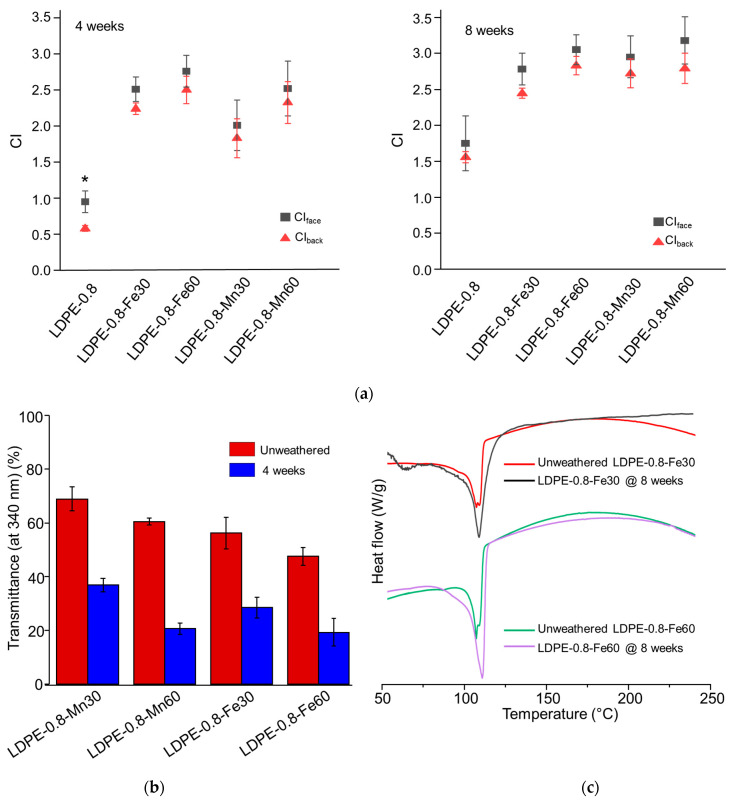
Impact of metal stearates on the photo-degradation of polyethylene sheets after 4 or 8 weeks of UV weathering. Polyethylene sheets containing single FeSt, single MnSt, or a combination of FeSt and MnSt were tested. The equivalent concentration of Fe or Mn in the polyethylene sheets was 30 ppm or 60 ppm. (**a**) CI_face_ and CI_back_ values of photo-oxidized polyethylene sheets. (**b**) UV transmittance of metal stearate-containing polyethylene sheets before and after 4 weeks of weathering. (**c**) DSC curves of FeSt-containing polyethylene sheets after 8 weeks of UV weathering. * indicates statistical significance (*p* < 0.05).

As a supplement to the ${\bar{M}}_{w}$ results, the CI detection results are shown in Figure 3a. Unlike in pristine LDPE-0.8, no significant difference between CI_face_ and CI_back_ was detected in metal stearate-containing polyethylene sheets after either 4 or 8 weeks of weathering. In comparisons of Fe and Mn at equivalent concentrations, LDPE-0.8-Mn30 exhibited a smaller CI_face_ value than did LDPE-0.8-Fe30 after 4 weeks of weathering, although this difference became less significant after 8 weeks. Previous studies [39,40] revealed that FeSt is efficient in inducing photo-oxidation, while MnSt is more competent in accelerating thermal oxidation. The results from this study are consistent with those of earlier research [26,41] that observed the rapid development of the CI during the first 4 weeks of photo-degradation, followed by a reduced growth rate for the CI until week 8. The development of the CI exhibited a synchronized time dependence in relation to the decrease in molecular weight, in which a plateau was observed. These results suggested a potential barrier that limited the further pro-oxidant effect of the metal stearates.

The UV transmittance of the metal stearate-containing LDPE sheets is characterized in Figure 3b. For the unweathered samples, the transmittance at 340 nm was detected as 56.2% ± 5.8% for LDPE-0.8-Fe30 and 47.5% ± 3.3% for LDPE-0.8-Fe60. In contrast, the reduction in UV transmittance for MnSt-containing LDPE sheets was minor compared to that for FeSt-containing samples. The transmittance at 340 nm was detected as 68.9% ± 4.4% for LDPE-0.8-Mn30 and 60.5% ± 1.3% for LDPE-0.8-Mn60, close to that for pristine LDPE-0.8 (70.6% ± 5.9%). After UV weathering, a significant decrease in UV transmittance was observed in both FeSt- and MnSt-containing samples, exemplified by a transmittance of 28.5% ± 3.9% detected in LDPE-0.8-Fe30 at week 4. Considering the accelerated degradation rate (Table 4), although FeSt reduced the initial UV transmittance of the LDPE sheet, the pro-oxidant effect was not mitigated at the tested Fe concentrations (30 and 60 ppm).

The crystallinity of the photo-oxidized metal stearate-containing LDPE sheets is summarized in Table 4. Unweathered FeSt- or MnSt-containing LDPE sheets exhibited a similar level of *Xc* when compared to pristine LDPE-0.8. After 4 and 8 weeks of UV weathering, all FeSt- and MnSt-containing LDPE sheets demonstrated increased *Xc* values. Specifically, LDPE-0.8-Fe60 exhibited an accelerated increase in *Xc* (83.2%) at week 4 compared to LDPE-0.8-Fe30 (74.8%) and pristine LDPE-0.8 (70.6%) (Table 3). The increase in *Xc* was statistically significant at week 8 when compared to unweathered samples. These results suggested that metal pro-oxidants could accelerate chemi-crystallization in LDPE sheets. The increase in crystallinity was corroborated by the DSC characterization in Figure 3c, which indicated that the melting peak area increased in LDPE-0.8-Fe30 and LDPE-0.8-Fe60 after 8 weeks of weathering. Furthermore, a characterization of oxygen transport was conducted, including the oxygen permeability (P*ox*), diffusivity (D) and solubility (S) (Table S1, Supporting Information). In the unweathered samples, the incorporation of FeSt or MnSt did not affect the Fickian coefficients for oxygen transport, as compared to those for pristine LDPE-0.8. Due to the development of cracks and significant embrittlement, equilibrium oxygen flux was not detected in the photo-oxidized metal stearate-containing LDPE sheets, except for LDPE-0.8-Mn30. After 2 weeks of weathering, the P*ox* value of LDPE-0.8-Mn30 decreased from 4.60 × 10^−10^ to 3.05 × 10^−10^ ccSTP/cm-s-cmHg, and the diffusivity decreased from 5.93 × 10^−7^ to 2.72 × 10^−7^ cm^2^/s, following a similar trend to that observed in pristine LDPE sheets (Table S1, Supporting Information). The increased crystalline components that formed as a result of accelerated photo-oxidation could hinder oxygen accessibility. After 8 weeks of weathering, the enhanced crystalline barrier, along with the reduced UV transmittance, collectively prevented the further progression of photo-degradation in metal stearate-containing polyethylene sheets, in which ${\bar{M}}_{w}$ was not reduced to below 5000 Da.

The metal stearates (FeSt and MnSt) exhibited a concentration-dependent pro-oxidant effect in accelerating the photo-degradation of polyethylene sheets. Additional considerations for the formulation design of photo-degradable polyethylene could involve food contact safety, colorants (by interacting with the light transmittance) [42], stabilizers, etc. [43]. Most polyethylene commodity products are intended for food contact applications. When incorporating metal-based pro-oxidants into polyethylene products, it is essential to regulate the potential leaching of metal ions and adhere to specific release limits. The Council of Europe (CoE) released Resolution CM/Res (2013)9 [44], which provides a guideline for the safety of metals and alloys in food contact materials. Specific release limits (SRLs) are suggested by CoE for iron (40 mg/kg food), magnesium (1.8 mg/kg food), cobalt (0.02 mg/kg food), etc. In the formulation design of photo-degradable polyethylene, it is essential to gain a comprehensive understanding of how each component interacts and influences the photo-degradation rate, while ensuring balanced performance in terms of product appearance, quality, safety, shelf life, etc.

### 3.4. Effect of Foaming and Resin Types on the Photo-Degradation of Polyethylene Sheets

**Figure 4:**
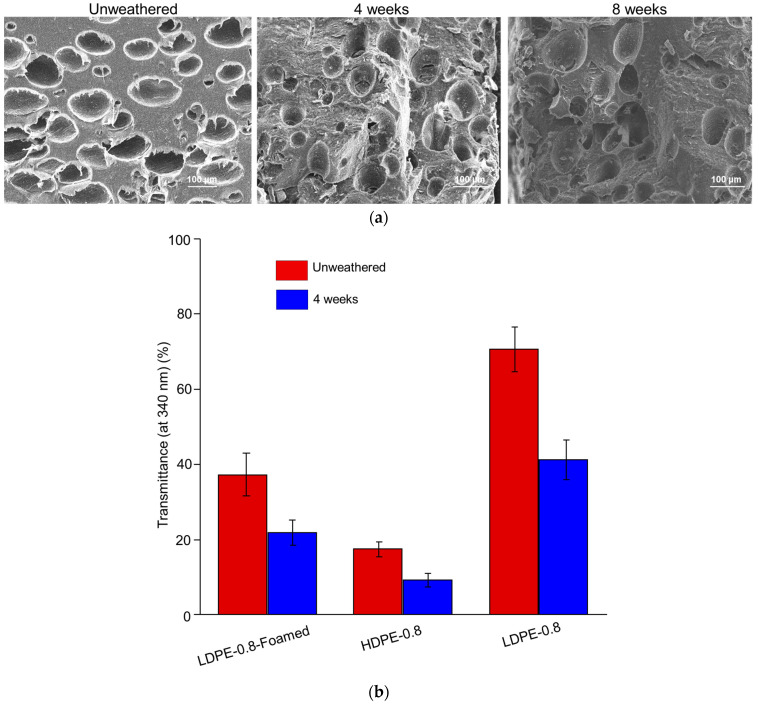

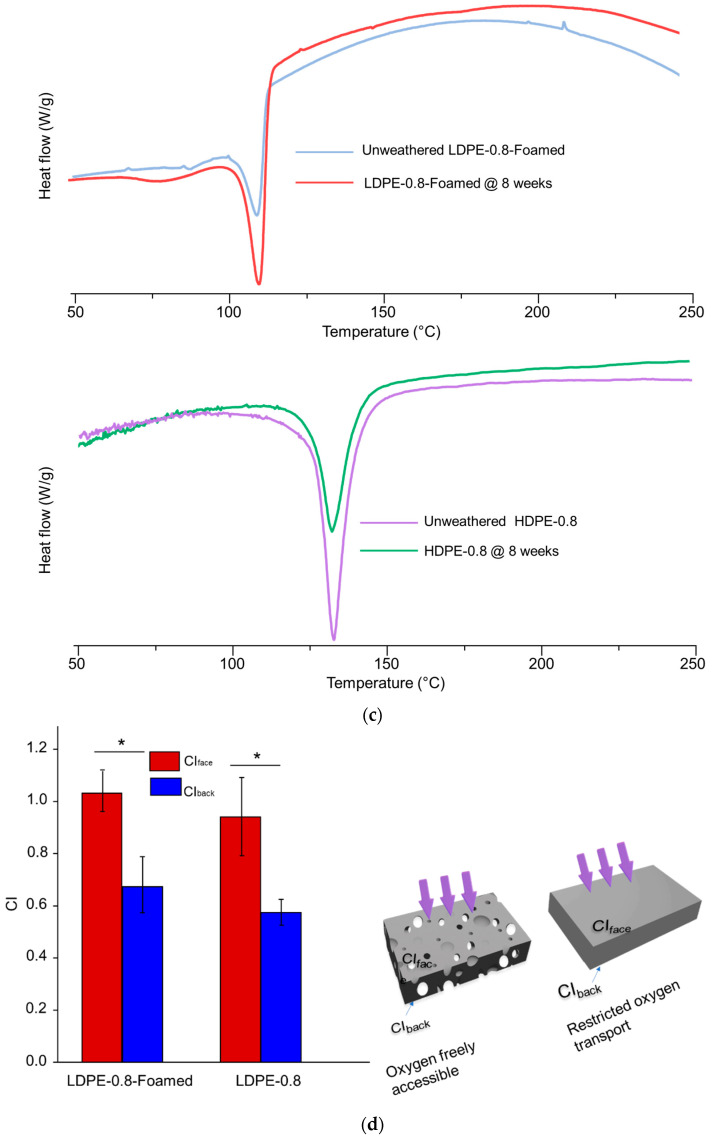
Impact of resin types and foaming on the photo-degradation of polyethylene sheets. (**a**) SEM morphological characterization of LDPE-0.8-Foamed after 4 and 8 weeks of UV weathering. (**b**) UV transmittance of LDPE-0.8-Foamed and HDPE-0.8 before and after 4 weeks of UV weathering in comparison with LDPE-0.8. (**c**) DSC characterization of LDPE-0.8-Foamed and HDPE-0.8 before and after 8 weeks of UV weathering. (**d**) After 4 weeks of UV weathering, CI values from unfoamed LDPE-0.8 and LDPE-0.8-Foamed were compared. * indicates statistical significance (*p* < 0.05).

To investigate the impact of the foaming structure on the photo-degradation of polyethylene sheets, Expancel blowing agent was incorporated with LDPE resin and extruded to prepare foamed LDPE sheets. The average size of the micropores in the foamed LDPE was approximately 100 μm. The morphological changes in and fragmentation of the foamed LDPE sheets after 4 and 8 weeks of UV weathering are shown in Figure 4a. As summarized in Table 5, the ${\bar{M}}_{w}$ value of LDPE-0.8-Foamed after either 4 or 8 weeks of weathering was greater than that of solid LDPE-0.8, indicating that foaming could potentially hinder photo-degradation. Furthermore, an HDPE-0.8 sheet was prepared from HDPE resin with a similar MFI (2 g/10 min). Although the initial ${\bar{M}}_{w}$ value of HDPE-0.8 was smaller than that of LDPE-0.8, the decrease in ${\bar{M}}_{w}$ in HDPE-0.8 after 4 or 8 weeks of weathering was significantly delayed (Table 5).

To correlate the optical properties with the photo-degradation rate, the UV transmittance of LDPE-0.8-Foamed and HDPE-0.8 is characterized in Figure 4b. Compared to that for LDPE-0.8, the UV transmittance for unweathered LDPE-0.8-Foamed (37.3% ± 5.6%) was significantly decreased, and this reduction was further amplified after 4 weeks of UV weathering (21.9% ± 3.3%). The introduction of a foaming structure rendered the unweathered LDPE sheet completely opaque due to the light scattering and reflection caused by the micropores. This obstruction of the trafficking of UV irradiation through LDPE-0.8-Foamed due to the presence of micropores could explain the delayed molecular weight decrease shown in Table 5. Additionally, HDPE-0.8 exhibited the lowest level of UV transmittance among all the tested samples (Figure 4b), with a transmittance of 9.2% ± 1.8% at 340 nm after 4 weeks of weathering, along with the most delayed molecular weight decrease.

The crystallinity was then characterized via DSC (Figure 4c) and WAXD. Compared to the LDPE sheet, a greater initial *Xc* value was detected in the unweathered HDPE sheet (81.4%), increasing to 87.2% after 8 weeks of weathering (Table 5). The highly crystalline characteristics of HDPE-0.8 are associated with impeded oxygen accessibility. The unweathered HDPE-0.8 exhibited significantly reduced P*ox* and D values when compared to all LDPE sheets, and this impedance was distinct even after 2 weeks of UV weathering (Table S3, Supporting Information). The restricted photo-oxidation rate in the HDPE sheet could be attributed to both reduced UV transmittance and restricted oxygen accessibility.

For the foamed LDPE sheet (LDPE-0.8-Foamed), the *Xc* value also displayed an increasing trend in the progress of photo-degradation (Table 5). This was evidenced by the increase in *Xc* from 63.2% (unweathered) to 75.8% after 8 weeks of weathering, which was statistically significant. It should be noted that the porous structure of LDPE-0.8-Foamed enhanced the accessibility of oxygen within the interior of the sample sheet. However, the rate of molecular weight decrease in LDPE-0.8-Foamed was slower than that in solid LDPE-0.8.

Furthermore, the photo-oxidation of foamed LDPE compared to that of its solid counterpart could indicate that the impact of UV transmittance was greater than that of oxygen accessibility. The micropores in LDPE-0.8-Foamed enhanced the availability of oxygen (Figure 4d). Meanwhile, the introduction of micropores reduced the transmittance at 340 nm by >40% compared to that in solid LDPE-0.8 (Figure 4b). The LDPE-0.8-Foamed sheet exhibited a reduced rate of molecular weight decrease during 8 weeks of UV weathering (Table 5), indicating that restricted UV penetration could have a more significant impact on the photo-oxidation rate than oxygen accessibility.

### 3.5. Detection of Chain Scission and Crosslinks in Photo-Oxidized Polyethylene Sheets

The concentrations of chain scission and crosslinks were calculated from the GPC detection results for ${\bar{M}}_{w}$ and ${\bar{M}}_{n}$ for all the polyethylene sheets after UV weathering [28]. By comparing the results for week 4 and week 8 (Table 6), the following trends were summarized. Firstly, growing concentrations of chain scission and crosslinks were evident with the progression of photo-oxidation. The occurrence of chain scission and crosslinks was negatively impacted by the increasing thickness, as indicated by a restricted growth rate in LDPE-1.2 compared to those in LDPE-0.8 and LDPE-0.4. Secondly, the incorporation of single metal stearates accelerated the development of chain scission and crosslinks in a concentration-dependent manner. In contrast, the porous structure of LDPE-0.8-Foamed and the greater initial crystallinity of HDPE-0.8 inhibited the development of chain scission and crosslinks.

**Figure 5:**
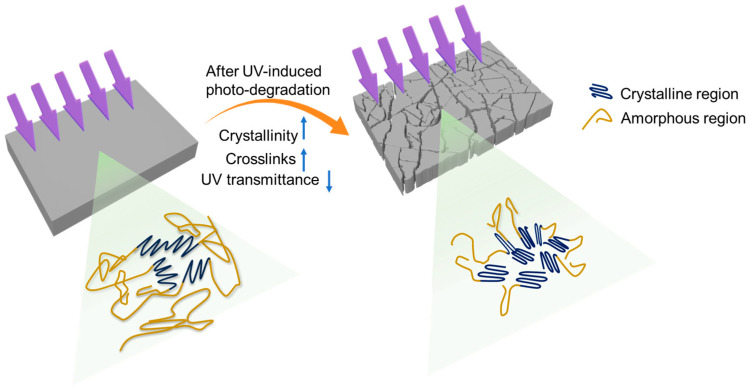
Schematic illustration of factors that hinder the further progression of photo-oxidation of polyethylene.

As illustrated in Figure 5, in the unweathered polyethylene, the crystallites were connected with each other by tie chains in the amorphous regions. During the progression of photo-degradation, chain scission mainly occurred within the amorphous regions, resulting in shorter polyethylene chains that facilitated chemi-crystallization. This increased crystallinity could account for the plateau phase without further molecular weight decrease or carbonyl development at the later stage of UV weathering (i.e., 8 weeks). In addition to increased crystallinity, the development of crosslinks enhanced chain immobilization and restricted the motility of polyethylene chains, thereby limiting oxygen transport. This trend explains the reduced oxygen accessibility in photo-oxidized polyethylene sheets (Table S1, Supporting Information) and could prevent the further propagation of photo-oxidation in polyethylene sheets.

### 3.6. Fungal Attachment and Degradation of Photo-Oxidized Polyethylene Sheets

**Figure 6:**
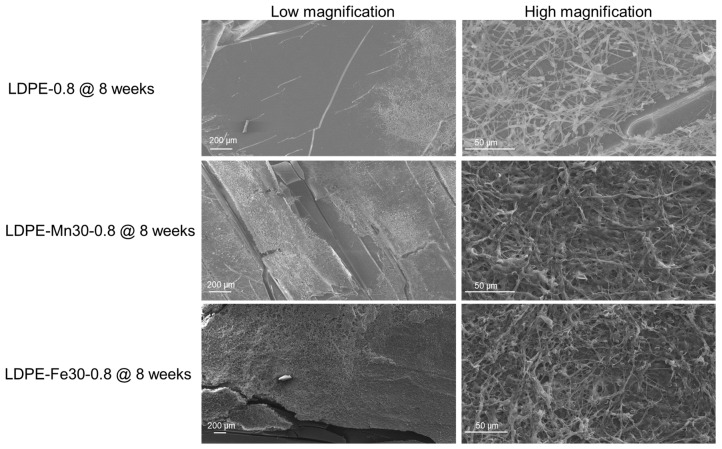
SEM characterization of the attachment of *P. chrysosporium* on the surface of photo-oxidized polyethylene sheets, including representative images of the morphology of fungal attachment on LDPE-0.8, LDPE-0.8-Fe30 and LDPE-0.8-Mn30 after 8 weeks of UV weathering followed by incubation with *P. chrysosporium* for 4 weeks.

The photo-oxidized polyethylene sheets were then incubated with *Phanerochaete chrysosporium*, a white-rot fungus commonly found in waste-composting systems. The extracellular release of manganese peroxidase (MnP) and laccase by *P. chrysosporium* could facilitate the further oxidation and degradation of polyethylene [45,46]. Polyethylene sheets after 4 weeks (LDPE-0.8 @ 4 weeks, LDPE-0.8-Fe30 @ 4 weeks and LDPE-0.8-Mn30 @ 4 weeks) or 8 weeks (LDPE-0.8 @ 8 weeks, LDPE-0.8-Fe30 @ 8 weeks and LDPE-0.8-Mn30 @ 8 weeks) of UV weathering were incubated with *P. chrysosporium* for an additional 4 weeks. The polyethylene sheets post incubation were characterized by SEM, and representative images of fungal attachment are shown in Figure 6. *P. chrysosporium* grew extensively to form compact biofilms on the surfaces of oxidized LDPE-0.8-Fe30 @ 8 weeks and LDPE-0.8-Mn30 @ 8 weeks. The formation of a biofilm on the plastic surface is considered a preliminary step in polymer biodegradation, as it enhances the accessibility of microbial cells and extracellular enzymes for the degradative attack of polymers. On the surface of LDPE-0.8, a less aggressive attachment of *P. chrysosporium* was observed, characterized by relatively loose bundles of hyphae. The attachment of *P. chrysosporium* on oxidized LDPE-0.8 @ 8 weeks was less uniform than that on metal stearate-containing LDPE sheets.

As compared to LDPE sheets without UV weathering, weight loss and a decrease in molecular weight were detected in photo-oxidized polyethylene sheets after incubation with *P. chrysosporium* (Table 7). No weight loss was detected in unweathered LDPE sheets, while minor weight loss after fungal incubation was detected in UV-weathered LDPE sheets (Table 7). LDPE-0.8-Mn30 @ 4 weeks and LDPE-0.8 @ 4 weeks both exhibited reduced ${\bar{M}}_{w}$ values and a narrowing of their molecular weight distributions, suggesting potential microbial degradation. Reduced ${\bar{M}}_{w}$ values were also observed in LDPE-0.8-Fe30 @ 4 weeks. However, in the case of LDPE sheets after 8 weeks of weathering, both LDPE-0.8 @ 8 weeks and LDPE-0.8-Fe30 @ 8 weeks were detected to have increased ${\bar{M}}_{w}$ values after fungal incubation. An increase in ${\bar{M}}_{w}$ was reported by Lee et al. [47] in their study of metal- and starch-containing polyethylene films incubated with *P. chrysosporium* and could be related to the further formation of chain crosslinks during biodegradation.

The above results indicate that photo-degradation accelerated by metal pro-oxidants could introduce more polar functional groups, including carbonyls (Table 2 and Figure S1, Supporting Information), onto the surface of polyethylene, thereby facilitating the anchoring and development of biofilms. However, it is hypothesized that the increased crystallinity in photo-oxidized polyethylene could hinder the degradative action of *P. chrysosporium.* Previous research [8] suggested that the crystalline region of polyethylene is less accessible to fungal enzymes than the amorphous region. When *P. chrysosporium* are extensively attached to photo-oxidized LDPE sheets, the high crystalline content (initial *Xc* values ranging from 70.6% to 87.5%, Table 3 and Table 4) could function as a biodegradation barrier. Santo et al. [48] reported an increase from 3.9% to 7.1% in *Xc* after 30 days of microbial biodegradation, indicating the persistence of this crystalline barrier. The fungal degradation results suggest that a balance should be sought by attaining a proper level of oxidized functional groups on the surface of polyethylene to support microbial attachment, while simultaneously maintaining a limited degree of crystallinity to enable effective biodegradative attack.

## 4. Conclusions

In this study, the photo-degradation process of polyethylene sheets was investigated under ASTM D5208-14 UV weathering conditions. The photo-degradation profile of the polyethylene sheets was evaluated by exploring the UV transmittance, tensile properties, molecular weight decrease, carbonyl development and changes in crystallinity. Factors such as thickness, metal pro-oxidants, polyethylene resin types and foaming were demonstrated to have a significant impact on the photo-degradation. Notable CI developments, molecular weight decreases and embrittlement were detected, especially in metal stearate-containing LDPE samples. Although the oxidized polyethylene surface facilitated the attachment and development of biofilms, the highly crystallized polyethylene sheets after 8 weeks of UV weathering were not sufficiently responsive to degradative attack by *P. chrysosporium*. In the multi-tiered degradation of polyethylene (photo-oxidation followed by biodegradation), it is crucial to address the barriers that restrict the progression of photo-oxidation to enhance biodegradative action. These barriers include delayed and heterogeneous degradation in thick polyethylene sheets, reduced UV transmittance that limits the activation of photo-oxidation, and an increased crystalline barrier during the photo-degradation process. Addressing these challenges could be essential for the formulation of photo-degradable polyethylene to facilitate the bio-assimilation of polyethylene waste.

## Figures and Tables

**Table 1:** List of formulations and abbreviations of polyethylene sheets investigated in this study.

Abbreviation	Formulation				
	Resin	Fe *	Mn *	Thickness	Blow Agent
LDPE-0.4	LDPE	0	0	0.4 mm	0
LDPE-0.8				0.8 mm	
LDPE-1.2				1.2 mm	
LDPE-0.8-Fe30	LDPE	30 ppm	0	0.8 mm	0
LDPE-0.8-Fe60		60 ppm			
LDPE-0.8-Mn30	LDPE		30 ppm	0.8 mm	0
LDPE-0.8-Mn60			60 ppm		
LDPE-0.8-Foamed	LDPE	0	0	0.8 mm	2 wt%
HDPE-0.8	HDPE	0	0	0.8 mm	0

* Iron or manganese was introduced into polyethylene sheets via iron stearate (FeSt) or manganese stearate (MnSt). The concentration of Fe or Mn is expressed in ppm (equivalent to mg of metal per kg of polyethylene).

**Table 2:** Carbonyl index and molecular weight characterization of polyethylene sheets in the time course of photo-degradation.

Duration of Weathering	LDPE-0.8					LDPE-0.8-Fe30				
	CI_face_	CI_back_	${\bar{M}}_{w}$ (Da)	${\bar{M}}_{w}$ Decrease (%)	Đ	CI_face_	CI_back_	${\bar{M}}_{w}$ (Da)	${\bar{M}}_{w}$ Decrease (%)	Đ
Unweathered	N.D. *		2.09 × 10^5^	0	5.93	N.D. *		2.07 × 10^5^	0	6.18
2 weeks	0.21 ± 0.08	0.18 ± 0.07	5.14 × 10^4^	75.36	7.18	1.49 ± 0.20	1.26 ± 0.12	3.08 × 10^4^	85.13	4.65
4 weeks	0.95 ± 0.15	0.58 ± 0.05	3.25 × 10^4^	84.42	6.94	2.51 ± 0.17	2.24 ± 0.08	1.32 × 10^4^	93.64	4.48
6 weeks	1.22 ± 0.23	0.98 ± 0.16	1.85 × 10^4^	91.14	4.73	2.67 ± 0.35	2.39 ± 0.25	5.63 × 10^3^	97.28	4.73
8 weeks	1.75 ± 0.38	1.60 ± 0.28	1.45 × 10^4^	93.07	5.12	2.78 ± 0.22	2.45 ± 0.07	5.44 × 10^3^	97.37	4.25

* No carbonyl peaks were detected in the ATR-FTIR spectra of unweathered polyethylene sheets.

**Table 3:** Molecular weight and crystallinity characterization of photo-oxidized polyethylene sheets with different thickness.

Sample	Unweathered				4 Weeks				8 Weeks			
	${\bar{M}}_{w}$ (Da)	${\bar{M}}_{w}$ Decrease (%)	Đ	*Xc* (%)	${\bar{M}}_{w}$ (Da)	${\bar{M}}_{w}$ Decrease (%)	Đ	*Xc* (%)	${\bar{M}}_{w}$ (Da)	${\bar{M}}_{w}$ Decrease (%)	Đ	*Xc* (%)
LDPE-0.4	2.07 × 10^5^	0	6.38	67.5 ± 5.9	1.53 × 10^4^	92.63	5.41	73.5 ± 6.2	7.55 × 10^3^	96.35	5.72	85.9 ± 5.4 *
LDPE-0.8	2.09 × 10^5^	0	5.93	64.3 ± 8.1	3.25 × 10^4^	84.42	6.94	70.6 ± 9.9	1.45 × 10^3^	93.07	5.12	85.2 ± 6.6 *
LDPE-1.2	2.07 × 10^5^	0	6.87	54.6 ± 9.2	8.63 × 10^4^	58.26	5.22	61.7 ± 8.8	3.75 × 10^3^	81.84	7.02	80.4 ± 4.2 *

* indicates that the X*c* value for the photo-oxidized samples was statistically significantly different to that for the unweathered samples.

**Table 4:** Molecular weight and crystallinity characterization of photo-oxidized polyethylene sheets with metal stearates.

Sample	Unweathered				4 Weeks				8 Weeks			
	${\bar{M}}_{w}\;(\mathrm{Da})$	${\bar{M}}_{w}$ Decrease (%)	Đ	*Xc* (%)	${\bar{M}}_{w}\;(\mathrm{Da})$	${\bar{M}}_{w}$ Decrease (%)	Đ	*Xc* (%)	${\bar{M}}_{w}\;(\mathrm{Da})$	${\bar{M}}_{w}$ Decrease (%)	Đ	*Xc* (%)
LDPE-0.8	2.09 × 10^5^	0	5.93	64.3 ± 8.1	3.25 × 10^4^	84.42	6.94	70.6 ± 9.9	1.45 × 10^3^	93.07	5.12	85.2 ± 6.6 *
LDPE-0.8-Fe30	2.07 × 10^5^	0	6.18	67.5 ± 1.2	1.32 × 10^4^	93.64	4.48	74.8 ± 3.9	5.44 × 10^3^	97.37	4.25	87.5 ± 4.1 *
LDPE-0.8-Fe60	1.75 × 10^5^	0	5.62	64.7 ± 5.3	8.92 × 10^3^	94.90	5.39	83.2 ± 8.5 *	5.05 × 10^3^	97.10	5.78	88.9 ± 6.2 *
LDPE-0.8-Mn30	2.04 × 10^5^	0	4.82	65.4 ± 8.1	2.65 × 10^4^	86.97	6.23	77.5 ± 6.9	1.23 × 10^4^	93.98	5.19	81.4 ± 2.8 *
LDPE-0.8-Mn60	2.00 × 10^5^	0	5.03	65.9 ±7.5	2.21 × 10^4^	88.91	5.36	80.2 ± 6.2 *	1.02 × 10^4^	94.89	6.56	82.5 ± 7.7 *

* indicates that the *Xc* value for photo-oxidized samples was statistically significantly different to that for unweathered samples.

**Table 5:** Molecular weight characterization of photo-oxidized polyethylene sheets with different resin types and foaming.

Sample	Unweathered				4 Weeks				8 Weeks			
	${\bar{M}}_{w}\;(\mathrm{Da})$	${\bar{M}}_{w}$ Decrease (%)	Đ	*Xc* (%)	${\bar{M}}_{w}\;(\mathrm{Da})$	${\bar{M}}_{w}$ Decrease (%)	Đ	*Xc* (%)	${\bar{M}}_{w}\;(\mathrm{Da})$	${\bar{M}}_{w}$ Decrease (%)	Đ	*Xc* (%)
LDPE-0.8	2.09 × 10^5^	0	5.93	64.3 ± 8.1	3.25 × 10^4^	84.42	6.94	70.6 ± 9.9	1.45 × 10^4^	93.07	5.12	85.2 ± 6.6 *
LDPE-0.8-Foamed	2.07 × 10^5^	0	5.87	63.2 ± 5.9	3.53 × 10^4^	82.99	5.06	64.9 ± 4.2	1.73 × 10^4^	91.66	4.02	75.8 ± 4.6
HDPE-0.8	1.05 × 10^5^	0	4.52	81.4 ± 1.4	6.19 × 10^4^	41.17	2.73	83.2 ± 2.8	2.71× 10^4^	74.24	6.24	87.2 ± 1.9 *

* indicates that the *Xc* value for photo-oxidized samples was statistically significantly different to that for unweathered samples.

**Table 6:** Changes in the concentrations of chain scission and crosslinks in polyethylene samples after 4 and 8 weeks of UV weathering.

Sample	Crosslinks (mol/kg)		Chain Scission (mol/kg)	
	4 Weeks	8 Weeks	4 Weeks	8 Weeks
LDPE-0.4	0.067	0.391	0.157	0.884
LDPE-0.8	0.044	0.230	0.066	0.391
LDPE-1.2	0.005	0.032	0.037	0.191
LDPE-0.8-Fe30	0.056	0.367	0.131	0.882
LDPE-0.8-Fe60	0.120	0.692	0.242	1.354
LDPE-0.8-Mn30	0.049	0.260	0.082	0.482
LDPE-0.8-Mn60	0.046	0.263	0.144	0.762
LDPE-0.8-Foamed	0.023	0.138	0.033	0.237
HDPE-0.8	0.006	0.035	0.044	0.231

**Table 7:** Detection of molecular weight and weight loss of photo-oxidized polyethylene sheets after fungal incubation for 4 weeks.

Sample	Before Incubation		After Incubation		Weight Loss (%)
	${\bar{M}}_{w}\;(\mathrm{Da})$	Đ	${\bar{M}}_{w}\;(\mathrm{Da})$	Đ	
LDPE-0.8 @ 4 weeks	3.25 × 10^4^	6.94	2.86 × 10^4^	5.92	1.05 ± 0.18
LDPE-0.8-Fe30 @ 4 weeks	1.32 × 10^4^	4.48	1.10 × 10^4^	4.92	3.95 ± 0.52
LDPE-0.8-Mn30 @ 4 weeks	2.65 × 10^4^	6.23	1.27 × 10^4^	4.24	1.87 ± 0.32
LDPE-0.8 @ 8 weeks	1.45 × 10^4^	5.12	2.75 × 10^4^	6.02	1.42 ± 0.14
LDPE-0.8-Fe30 @ 8 weeks	5.44 × 10^3^	4.25	1.10 × 10^4^	5.68	1.72 ± 0.39
LDPE-0.8-Mn30 @ 8 weeks	1.23 × 10^4^	5.19	1.03 × 10^4^	4.39	0.87 ± 0.33

## Data Availability

The data that support the findings of this study are available from the corresponding author upon reasonable request.

## Supplementary Materials

The following supporting information can be downloaded at: https://www.mdpi.com/article/10.3390/polym16192709/s1, Figure S1: Detection of UV transmittance at 340 nm during the time course of LDPE oxo-degradation under accelerated weathering; Figure S2: HT-GPC chromatographs of photo-oxidized LDPE sheets with different thicknesses; Figure S3: Depth-dependent profile of relative oxygen concentration (θ) and CI in photo-oxidized LDPE-1.2. Slices in 200 μm increments were sectioned from oxidized LDPE-1.2 after 2 or 4 weeks of UV weathering, with relative depth X = 0, 0.17, 0.33, 0.67, 0.83 and 1. CI characterization of microtome-sectioned slices was performed; θ was calculated and plotted versus X; Table S1: Impact of UV weathering on oxygen transport with different resin types and foaming, metal stearate-containing polyethylene sheets of three thicknesses; Table S2: Factors for the calculation of the relative oxygen concentration θi at different depths in slices of photo-oxidized LDPE-1.2 after 2 weeks of UV weathering; Table S3: Factors for the calculation of the relative oxygen concentration at different depths in slices of photo-oxidized LDPE-1.2 after 4 weeks of UV weathering. References [31,37,38,49,50,51] appear in the Supplementary Materials file.

## References

[1] Bodzay B., Bánhegyi G. (2016). Polymer waste: Controlled breakdown or recycling?. Int. J. Des. Sci. Technol..

[2] Singh N., Hui D., Singh R., Ahuja I., Feo L., Fraternali F. (2017). Recycling of plastic solid waste: A state of art review and future applications. Compos. Part B Eng..

[3] Peng Y., Wu P., Schartup A.T., Zhang Y. (2021). Plastic waste release caused by COVID-19 and its fate in the global ocean. Proc. Natl. Acad. Sci. USA.

[4] Awaja F., Pavel D. (2005). Recycling of PET. Eur. Polym. J..

[5] Fiorente A., D’Agostino G., Petrella A., Todaro F., Notarnicola M. (2024). Recovery of Plastics from WEEE through Green Sink–Float Treatment. Materials.

[6] Vanapalli K.R., Sharma H.B., Ranjan V.P., Samal B., Bhattacharya J., Dubey B.K., Goel S. (2021). Challenges and strategies for effective plastic waste management during and post COVID-19 pandemic. Sci. Total Environ..

[7] Environmental Protection Agency (2020). Advancing Sustainable Materials Management: 2018 Fact Sheet. Assessing Trends in Materials Generation and Management in the United States.

[8] Restrepo-Flórez J.-M., Bassi A., Thompson M.R. (2014). Microbial degradation and deterioration of polyethylene—A review. Int. Biodeterior. Biodegrad..

[9] Bahl S., Dolma J., Singh J.J., Sehgal S. (2021). Biodegradation of plastics: A state of the art review. Mater. Today Proc..

[10] Koutny M., Lemaire J., Delort A.-M. (2006). Biodegradation of polyethylene films with prooxidant additives. Chemosphere.

[11] (2021). Standard Guide for Exposing and Testing Plastics That Degrade in the Environment by a Combination of Oxidation and Biodegradation.

[12] Sen S.K., Raut S. (2015). Microbial degradation of low density polyethylene (LDPE): A review. J. Environ. Chem. Eng..

[13] Gardette M., Perthue A., Gardette J.-L., Janecska T., Földes E., Pukánszky B., Therias S. (2013). Photo-and thermal-oxidation of polyethylene: Comparison of mechanisms and influence of unsaturation content. Polym. Degrad. Stab..

[14] Ammala A., Bateman S., Dean K., Petinakis E., Sangwan P., Wong S., Yuan Q., Yu L., Patrick C., Leong K. (2011). An overview of degradable and biodegradable polyolefins. Prog. Polym. Sci..

[15] Abrusci C., Pablos J.L., Marín I., Espí E., Corrales T., Catalina F. (2013). Comparative effect of metal stearates as pro-oxidant additives on bacterial biodegradation of thermal-and photo-degraded low density polyethylene mulching films. Int. Biodeterior. Biodegrad..

[16] Monkul M.M., Özhan H.O. (2021). Microplastic contamination in soils: A review from geotechnical engineering view. Polymers.

[17] Therias S., Rapp G., Masson C., Gardette J.-L. (2021). Limits of UV-light acceleration on the photooxidation of low-density polyethylene. Polym. Degrad. Stab..

[18] Antunes M.C., Agnelli J.A., Babetto A.S., Bonse B.C., Bettini S.H. (2017). Abiotic thermo-oxidative degradation of high density polyethylene: Effect of manganese stearate concentration. Polym. Degrad. Stab..

[19] Chiellini E., Corti A., Swift G. (2003). Biodegradation of thermally-oxidized, fragmented low-density polyethylenes. Polym. Degrad. Stab..

[20] Fa W., Wang J., Ge S., Chao C. (2020). Performance of photo-degradation and thermo-degradation of polyethylene with photo-catalysts and thermo-oxidant additives. Polym. Bull..

[21] Benítez A., Sánchez J.J., Arnal M.L., Müller A.J., Rodríguez O., Morales G. (2013). Abiotic degradation of LDPE and LLDPE formulated with a pro-oxidant additive. Polym. Degrad. Stab..

[22] Roé-Sosa A., Estrada M.R., Calderas F., Sánchez-Arévalo F., Manero O., de Velasquez M.T.O.L. (2015). Degradation and biodegradation of polyethylene with pro-oxidant aditives under compost conditions establishing relationships between physicochemical and rheological parameters. J. Appl. Polym. Sci..

[23] (2021). Standard Practice for Fluorescent Ultraviolet (UV) Exposure of Photodegradable Plastics.

[24] (2021). Standard Test Method for Tensile Properties of Plastics.

[25] (2021). Standard Practice for Determining Degradation End Point in Degradable Polyethylene and Polypropylene Using a Tensile Test.

[26] Almond J., Sugumaar P., Wenzel M.N., Hill G., Wallis C. (2020). Determination of the carbonyl index of polyethylene and polypropylene using specified area under band methodology with ATR-FTIR spectroscopy. e-Polymers.

[27] Mo Z., Zhang H. (1995). The degree of crystallinity in polymers by wide-angle x-ray diffraction (WAXD). J. Macromol. Sci. Part C Polym. Rev..

[28] Fayolle B., Colin X., Audouin L., Verdu J. (2007). Mechanism of degradation induced embrittlement in polyethylene. Polym. Degrad. Stab..

[29] (2021). Plastics—Evaluation of the Action of Microorganisms.

[30] Kuka E., Cirule D., Andersone I., Vasiljevs L.O., Merna J., Sarakovskis A., Kurnosova N., Sansonetti E., Vevere L., Andersons B. (2024). A step to microplastic formation: Microcracking and associated surface transformations of recycled LDPE, LLDPE, HDPE, and PP plastics exposed to UV radiation. Polym. Degrad. Stab..

[31] Rodriguez A.K., Mansoor B., Ayoub G., Colin X., Benzerga A.A. (2020). Effect of UV-aging on the mechanical and fracture behavior of low density polyethylene. Polym. Degrad. Stab..

[32] Hsu Y.-C., Weir M.P., Truss R.W., Garvey C.J., Nicholson T.M., Halley P.J. (2012). A fundamental study on photo-oxidative degradation of linear low density polyethylene films at embrittlement. Polymer.

[33] Andrady A., Pegram J., Tropsha Y. (1993). Changes in carbonyl index and average molecular weight on embrittlement of enhanced-photodegradable polyethylenes. J. Environ. Polym. Degrad..

[34] Gulmine J., Janissek P., Heise H., Akcelrud L. (2003). Degradation profile of polyethylene after artificial accelerated weathering. Polym. Degrad. Stab..

[35] Moreira C., Lloyd R., Hill G., Huynh F., Trufasila A., Ly F., Sawal H., Wallis C. (2021). Temperate UV-accelerated weathering cycle combined with HT-GPC analysis and drop point testing for determining the environmental instability of polyethylene films. Polymers.

[36] Fayolle B., Richaud E., Colin X., Verdu J. (2008). Degradation-induced embrittlement in semi-crystalline polymers having their amorphous phase in rubbery state. J. Mater. Sci..

[37] Quintana A., Celina M.C. (2018). Overview of DLO modeling and approaches to predict heterogeneous oxidative polymer degradation. Polym. Degrad. Stab..

[38] Cunliffe A., Davis A. (1982). Photo-oxidation of thick polymer samples—Part II: The influence of oxygen diffusion on the natural and artificial weathering of polyolefins. Polym. Degrad. Stab..

[39] Liu X., Gao C., Sangwan P., Yu L., Tong Z. (2014). Accelerating the degradation of polyolefins through additives and blending. J. Appl. Polym. Sci..

[40] Muthukumar T., Aravinthan A., Mukesh D. (2010). Effect of environment on the degradation of starch and pro-oxidant blended polyolefins. Polym. Degrad. Stab..

[41] Martínez-Romo A., González-Mota R., Soto-Bernal J., Rosales-Candelas I. (2015). Investigating the Degradability of HDPE, LDPE, PE-BIO, and PE-OXO Films under UV-B Radiation. J. Spectrosc..

[42] Maalihan R.D., Pajarito B.B. (2016). Effect of colorant, thickness, and pro-oxidant loading on degradation of low-density polyethylene films during thermal aging. J. Plast. Film Sheeting.

[43] Focke W.W., Mashele R.P., Nhlapo N.S. (2011). Stabilization of low-density polyethylene films containing metal stearates as photodegradants. J. Vinyl Addit. Technol..

[44] Council of Europe (2022). Resolution CM/Res(2013)9 on Metals and Alloys Used in Food Contact Materials and Articles.

[45] Mukherjee S., Kundu P.P. (2014). Alkaline fungal degradation of oxidized polyethylene in black liquor: Studies on the effect of lignin peroxidases and manganese peroxidases. J. Appl. Polym. Sci..

[46] Iiyoshi Y., Tsutsumi Y., Nishida T. (1998). Polyethylene degradation by lignin-degrading fungi and manganese peroxidase. J. Wood Sci..

[47] Lee B., Pometto A.L., Fratzke A., Bailey T.B. (1991). Biodegradation of degradable plastic polyethylene by Phanerochaete and Streptomyces species. Appl. Environ. Microbiol..

[48] Santo M., Weitsman R., Sivan A. (2013). The role of the copper-binding enzyme–laccase–in the biodegradation of polyethylene by the actinomycete Rhodococcus ruber. Int. Biodeterior. Biodegrad..

[49] Michaels A.S., Bixler H.J. (1961). Flow of gases through polyethylene. J. Polym. Sci..

[50] François-Heude A., Richaud E., Guinault A., Desnoux E., Colin X. (2015). Impact of Oxygen Transport Properties on Polypropylene Thermal Oxidation, Part 1: Effect of Oxygen Solubility. J. Appl. Polym. Sci..

[51] François-Heude A., Richaud E., Desnoux E., Colin X. (2015). A general kinetic model for the photothermal oxidation of polypropylene. J. Photochem. Photobiol. A Chem..

