# Supplementary material for: Study of Factors Affecting UV-Induced Photo-Degradation in Different Types of Polyethylene Sheets
Source: Polymers (Basel). 2024 Sep 25;16(19):2709. doi: 10.3390/polym16192709 (PMC11478657; doi:10.3390/polym16192709)
Supplement: Supplementary file 1 [file polymers-16-02709-s001.zip › polymers-3170639-supplementary.pdf]

Supplementary Materials

Study of factors affecting UV-induced photo-degradation in different types of polyethylene sheets

Bochu Du, Chenghao Lee, Ying Ji*
Research Centre for Resources Engineering Towards Carbon Neutrality, Research Institute for Intelligent Wearable Systems, Hong Kong Polytechnic University, Kowloon, Hong Kong SAR, China

Detection of UV transmittance and molecular weight over the time course of UV weathering

The calculation of carbonyl index (CI) was performed via the specified area under band (SAUB) method, by comparing the peak area of total carbonyls (1670 to 1850 cm^{-1}) to the reference area of methylene scissoring peak at 1420 to 1500 cm^{-1}. As shown in Figure S1, the transmittance of UV irradiation at 340nm was detected as 71.8% for unweathered LDPE-0.8. With the progression of photo-oxidation, the UV transmittance at 340 nm reduced accordingly. For example, the transmittance of LDPE-0.8 reduced to 46.7% after 4 weeks of weathering, which indicated the reduced accessibility of UV irradiation at the back side of the samples.

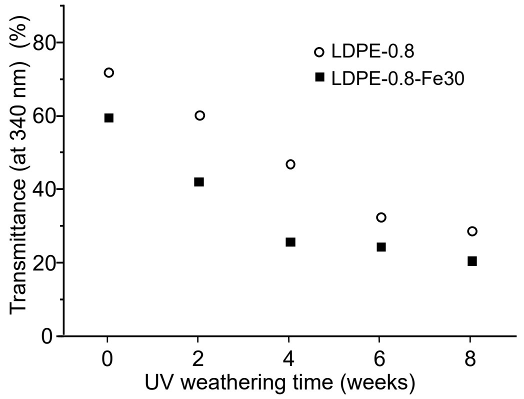

Figure S1. Detection of UV transmittance at 340 nm during the time course of LDPE oxo-degradation at accelerated weathering.

HT-GPC was performed on a 1260 Infinity II HT-GPC system (Agilent) with three PLgel MIXED-B LS (300 ×7.5mm) columns. Refractive index detector was utilized and the retention time of polyethylene samples were calibrated to polystyrene standards. A universal calibration was established between Log (MW*[η]) and retention time. The intrinsic viscosity [η] was calculated by Mark-Houwink equation ($[\eta] = KMW^{\alpha}$). As per manufacture's instruction, the parameters K, α for polymers in trichlorobenzene were: polystyrene $K = 12.1 \times 10^{-5}$ dL/g and $\alpha = 0.707$; polyethylene $K = 40.6 \times 10^{-5}$ dL/g and $\alpha = 0.725$. The molecular weight range of polyethylene that followed universal calibration was 300000 Da to 1300 Da. LDPE samples with different thicknesses were tested after 4 or 8 weeks of photo-oxidation.

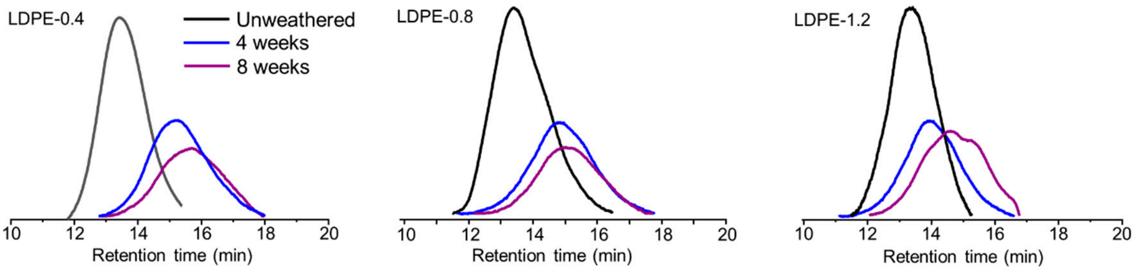

Figure S2. HT-GPC chromatographs of photo-oxidized LDPE sheets with different thicknesses.

Characterization of oxygen transport in different types of photo-oxidized polyethylene

The oxygen permeation was tested on a Mocon OX-TRAN 2/22-H coulometric oxygen permeation analyzer. Before and after UV weathering, the polyethylene samples were mounted with a sample size of 5.64 cm^2 and purged with nitrogen. The partial oxygen pressure at the feed side was fixed at 0.5 atm and the temperature was maintained at 25 °C. The oxygen permeation flux was monitored as a function of time until equilibrium was reached. The oxygen permeability (P_{ox}), diffusivity (D) and solubility (S) were determined based on the following equations.[49]

$$P_{ox} = \frac{Q}{(\frac{\Delta P}{L})};\ D = \frac{L^2}{6t};\ P_{ox} = DS$$

where Q was the permeation flux of oxygen at equilibrium, ΔP was the oxygen partial pressure, L was the sample thickness and t was the time-lag.

In addition to UV transmittance and crystallinity, the oxygen transport in polyethylene sheets with different thickness were characterized. Table S1 summarized the Fiskian coefficients including oxygen permeability (P_{ox}), diffusivity (D), and solubility (S) of polyethylene sheets before and after 2 weeks of UV weathering. It should be noted that with crack development which could lead to gas leakage, oxygen permeation test for polyethylene sheets photo-oxidized for longer than 2 weeks was not applicable due to the absence of equilibrium oxygen flux. Furthermore, after 4 to 8 weeks of UV weathering, the embrittled samples added difficulties to the oxygen permeation test. The P_{ox} of LDPE-0.8-Foamed was beyond the upper detection limit of the oxygen permeation analyzer, which could be a result of the presence of interconnected micropores. However, the rate of molecular weight decreases in LDPE-0.8-Foamed was delayed compared to solid LDPE-0.8. In the case of unweathered samples, Table S1 indicated that P_{ox} was inversely correlated with thickness. The diffusivity of unweathered LDPE-1.2 (9.52×10^{-7} cm^2/s) was greater than LDPE-0.4 and LDPE-0.8, which could be a result of the lower initial crystallinity of LDPE-1.2 (54.6%). After 2 weeks of weathering, both P_{ox} and D of all the samples were reduced (Table S1), in accordance with the increased X_c (Tables 3 to 5, main text). Being more accessible to oxidative attack, the amorphous polyethylene could be the primary target of photo-degradation and the degraded polyethylene could be subject to chemi-crystallization.[31] As photo-oxidation progressed, the increased crystalline components provided geometric impedance and restricted the motility of polyethylene chains. Therefore, the increased X_c dampened the oxygen accessibility in photo-oxidized polyethylene sheets which could restrict the oxygen supply for oxidative degradation.

Table S1. Impact of UV weathering on oxygen transport with different resin types and foaming, metal stearate containing and three thicknesses polyethylene sheets.

Samples	Oxygen permeability (ccSTP/cm-s-cmHg)		Oxygen diffusivity (cm^2/s)		Oxygen solubility (ccSTP/cm^3-cmHg)	
	Unweathered	2 weeks *	Unweathered	2 weeks *	Unweathered	2 weeks *
LDPE-0.8	4.25×10^{-10}	3.96×10^{-10}	6.35×10^{-7}	4.06×10^{-7}	6.72×10^{-4}	9.79×10^{-4}
LDPE-0.8-Foamed	$>2.10\times10^{-9}$	N.D.	N.D.	N.D.	N.D.	N.D.
HDPE-0.8	1.32×10^{-10}	8.17×10^{-11}	3.29×10^{-7}	2.42×10^{-7}	4.02×10^{-4}	3.37×10^{-4}
LDPE-0.8-Fe30	5.01×10^{-10}	N.D.	5.35×10^{-7}	N.D.	N.D.	N.D.
LDPE-0.8-Fe60	4.23×10^{-10}	N.D.	7.18×10^{-7}	N.D.	N.D.	N.D.
LDPE-0.8-Mn30	4.60×10^{-10}	3.05×10^{-10}	5.93×10^{-7}	2.72×10^{-7}	7.76×10^{-4}	11.23×10^{-4}
LDPE-0.8-Mn60	4.13×10^{-10}	N.D.	6.28×10^{-7}	N.D.	N.D.	N.D.
LDPE-0.4	6.44×10^{-10}	5.27×10^{-10}	4.36×10^{-7}	2.46×10^{-7}	14.83×10^{-4}	21.5×10^{-4}
LDPE-0.8	4.25×10^{-10}	3.96×10^{-10}	6.35×10^{-7}	4.06×10^{-7}	6.72×10^{-4}	9.79×10^{-4}
LDPE-1.2	3.01×10^{-10}	2.59×10^{-10}	9.52×10^{-7}	6.89×10^{-7}	3.18×10^{-4}	3.78×10^{-4}

*The Fickian coefficients for oxygen transport in photo-oxidized metal stearate-containing polyethylene sheets were not detected (N.D.) due to the presence of cracks that prevented the equilibrium oxygen flux in the coulometric permeation test.

Analysis of the depth-dependent oxygen content in photo-oxidized LDPE-1.2

Previous research has demonstrated the importance of oxygen diffusion which contribute to the degradation rate of thick polymer samples.[50] The consumption of oxygen by the oxidative reaction could deplete the oxygen supply and result in a depth-dependent, heterogeneous degradation profile in thick samples. To perform a preliminary analysis of the spatial distribution of oxygen distribution in photo-oxidized LDPE-1.2, the following diffusion-limited oxidation analysis[4] is adopted to investigate the competing oxygen consumption and oxygen diffusion during the oxidation of polymers at a steady state.

$$\frac{d^2\theta}{dX^2} = \frac{\alpha\theta}{\beta\theta + 1}$$

in which X (0 to 1) is the relative depth, defined by the depth x (distance to the UV-irradiated face) divided by the total thickness L; θ (0 to 1) is the relative oxygen concentration defined by the oxygen partial pressure (P) at position x normalized by the oxygen partial pressure at the sample surface (P_0).

$$\alpha = \frac{k_1 L^2}{D}$$

k_1 is the initiation rate constant divided by the termination rate constant of oxidative reaction, and D represents the diffusivity of oxygen in the polymer sample.

$$\beta = S k_2 P_0$$

k_2 is the propagation rate constant divided by the termination rate constant of oxidative reaction, and S represents the solubility of oxygen.

In an attempt to explore the oxygen consumption versus oxygen diffusion in the UV-induced photo-oxidation of LDPE-1.2 after weathering, the following analysis was performed.

At a fixed photo-oxidation duration and temperature, the UV irradiance is dependent on X instead of being a constant. As demonstrated in Figure S1, the UV transmittance decreased when trafficking across the thick sample, which could result in a reducing k_1 (due to the reduced availability of UV irradiation to initiate the oxidation). Therefore, the heterogenous α is depth-dependent and could lead to an unsymmetrical profile of θ. As illustrated in Figure S2, when X=0, maximum photo-oxidation rate (oxygen consumption rate) and maximum oxygen accessibility is expected. When X=1, minimum oxygen consumption rate (due to the restricted UV transmittance) and maximum oxygen consumption rate is expected. This contrasted with the thermal oxidation of polyolefins, in which the initiation rate constant is dependent on temperature and could be uniform from X=0 to X=1. In an attempt to solve θ at the representative slices (slices in 200 μm increment were microtome-sectioned from oxidized LDPE-1.2, with X= 0.17, 0.67, and 1), the following analysis was performed.

To estimate β, the oxygen solubility of was determined as 3.18×10^{-4} ccSTP/cm^3-cmHg for unweathered LDPE-1.2 and 3.18×10^{-4} ccSTP/cm^3-cmHg for LDPE-1.2 @ 2 weeks (Table S1). The diffusivity and solubility were not detectable for LDPE-1.2 @ 4 weeks, therefore, we use the Fickian coefficients detected in unweathered LDPE-1.2 for the following analysis. The propagation rate constants for the photo and thermal

oxidation of polyolefins were reported in the range of 10^7 to 10^9 L/mol/s and the termination rate constants were reported ranging from 10^{10} to 10^{17} L/mol/s,[53, 54] which leads to k_2 «1. Therefore, $\beta\theta = Sk_2P_0\theta \ll 1$ and could be neglected. θ is primarily dependent on α.

$$\frac{d^2\theta}{dX^2} = \alpha\theta$$

At position X_i, θ could be expressed by solving the above differential equation:

$\theta_i = C_1 e^{\sqrt{\alpha_i}X_i} + C_2 e^{-\sqrt{\alpha_i}X_i}$, in which $\alpha = \frac{k_1L^2}{D} = \frac{R_0L^2}{DC_0}(1+\beta) \approx \frac{R_0L^2}{DSP_0}$

Based on the detected CI values of the slices (Figure S2), α_i could be estimated by calculating the average rate of oxygen absorption (R) after certain weathering time *t*, as reported by Cunliffe *et al.* [51]

$\alpha_i \approx \frac{R_iL^2}{DSP_0}$ and $R_i = \frac{0.174}{t} \times 41.9\ CI\ ccSTP/(cm^3s)$

The estimation of α_i was performed to LDPE-1.2 after 2 or 4 weeks of UV weathering and θ_i was further calculated by solving the constants C_1 and C_2 by the boundary conditions (X=0, θ=1; X=1, θ=1) and listed in Tables S2 and S3. The calculated θ_i was plotted in Figure S2. The trend of θ versus X demonstrated an unsymmetrical profile, that the minimum θ occurred in positions above the central layer of the sample (X<0.5), which differentiated with the symmetrical profile in which $\frac{d\theta}{dX} = 0$ applied to the central layer (X=0.5). As shown in Figure S2, the distribution of oxygen concentration exhibited an unsymmetrical profile in which maximum oxygen concentration was identified at both upper face and back side, while the minimum θ occurred in position above the central layer of the sample (X<0.5). The unsymmetrical distribution of θ could be correlated with the heterogenous photo-initiation rate, as a result of the thickness-dependent UV transmittance in Figure 2C (main text). In addition, greater depth-dependency of θ was indicated at week 4 compared to week 2, which could be a result of the enhanced oxidation rate and the impact of oxygen consumption was greater as the photo-oxidation progressed.

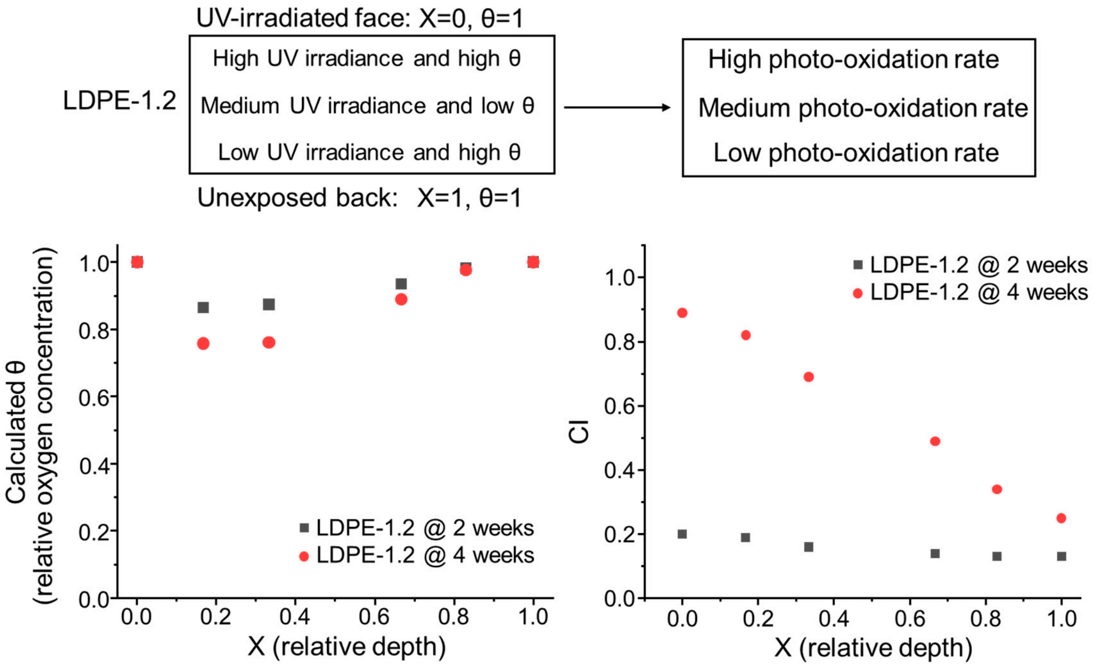

Figure S3. Depth-dependent profile of relative oxygen concentration (θ) and CI in photo-oxidized LDPE-1.2. Slices in 200 μm increment were sectioned from oxidized LDPE-1.2 after 2 or 4 weeks of UV weathering, with the relative depth X= 0, 0.17, 0.33, 0.67, 0.83 and 1. CI characterization of microtome-sectioned slices was performed, θ was calculated and plotted versus X.

Table S2. Factors for the calculation of relative oxygen concentration θi at different depths in the slices of photo-oxidized LDPE-1.2 after 2 weeks of UV weathering.

X_i	Distance to face in mm	Distance to back in mm	CI	R_i ccSTP/(cm^{3}s)	α_i	C_1	C_2	θi
0	0	1.2	0.20	12.05×10^{-7}	3.60	0.13	0.87	1.00
0.17	0.2	1.0	0.19	11.44×10^{-7}	2.38	0.18	0.82	0.86
0.33	0.4	0.8	0.16	9.64×10^{-7}	1.28	0.24	0.76	0.87
0.67	0.6	0.6	0.14	8.43×10^{-7}	0.63	0.31	0.69	0.93
0.83	0.8	0.4	0.13	7.83×10^{-7}	0.26	0.38	0.62	0.98
1	1.0	0.2	0.13	7.83×10^{-7}	0.07	0.44	0.56	1.00

Table S3. Factors for the calculation of relative oxygen concentrate at different depths in the slices of photo-oxidized LDPE-1.2 after 4 weeks of UV weathering.

X_i	Distance to face in mm	Distance to back in mm	CI	R_i ccSTP/(cm^3s)	α_i	C_1	C_2	θi
0	0	1.2	0.89	26.81×10^{-7}	8.02	0.06	0.94	1.00
0.17	0.2	1.0	0.82	24.70×10^{-7}	5.13	0.09	0.91	0.76
0.33	0.4	0.8	0.69	20.78×10^{-7}	2.76	0.16	0.84	0.76
0.67	0.6	0.6	0.49	14.76×10^{-7}	1.10	0.26	0.74	0.89
0.83	0.8	0.4	0.34	10.24×10^{-7}	0.34	0.36	0.64	0.98
1	1.0	0.2	0.25	7.53×10^{-7}	0.06	0.44	0.56	1.00

When using CI as the indicator of oxidation progress, the profile of CI versus X deviated from the profile of θ versus X (Figure S2). The CI of photo-oxidized LDPE-1.2 slices exhibited a reducing trend and was inversely related with the depth. By comparing the profile of UV transmittance (Figure S1) and θ_i (Figure S2), the trend of oxidation progress versus depth in LDPE-1.2 demonstrated similarity with the UV transmittance profile instead of oxygen distribution. The result could indicate that the impact of UV availability at different depths of samples could potentially outweigh the impact of oxygen distribution at the early to middle stage of ASTM D5208 UV weathering condition (2 and 4 weeks). Future research efforts should focus on the comprehensive investigation of the reactive transport relationships of oxygen during the photo-oxidation of polyethylene sheets, with consideration of time course, temporal-spatial dynamic oxygen consumption rate, the impact of crack formation and crystallinity development, *etc.*

References

[31] A. K. Rodriguez, B. Mansoor, G. Ayoub, X. Colin, A. A. Benzerga. Polymer Degradation and Stability. **2020**, 180, 109185-109199.
[37] A. V. Cunliffe, A. Davis. Polymer Degradation and Stability. **1982**, 4, 17-37.
[38] A. Quintana, M. C. Celina. Polymer Degradation and Stability. **2018**, 149, 173-191.
[49] A. S. Michaels, H. J. Bixler. Journal of Polymer Science. **1961**, 50, 413-439.
[50] A. François-Heude, E. Richaud, A. Guinault, E. Desnoux, X. Colin. Journal of Applied Polymer Science. **2015**, 132, 41441.
[51] A. François-Heude, E. Richaud, E. Desnoux, X. Colin. Journal of Photochemistry and Photobiology. A, Chemistry. **2015**, 296, 48-65.
